# A pilot feasibility randomised controlled trial of two behaviour change interventions compared to usual care to reduce substance misuse in looked after children and care leavers aged 12-20 years: The SOLID study

**DOI:** 10.1371/journal.pone.0238286

**Published:** 2020-09-08

**Authors:** Hayley Alderson, Eileen Kaner, Elaine McColl, Denise Howel, Tony Fouweather, Ruth McGovern, Alex Copello, Heather Brown, Paul McArdle, Deborah Smart, Rebecca Brown, Raghu Lingam

**Affiliations:** 1 Population Health Sciences Institute, Newcastle University, Newcastle, United Kingdom; 2 School of Psychology, University of Birmingham, Birmingham, United Kingdom; 3 Child and Adolescent, Mental Health Services, Northumberland, Tyne and Wear NHS Foundation Trust, Newcastle, United Kingdom; 4 Population Child Health Research Group, School of Women and Children’s Health, University New South Wales, Randwick, Australia; University College London, UNITED KINGDOM

## Abstract

**Background:**

Young people in state care, often due to abuse or neglect, have a four-fold increased risk of drug and alcohol use compared to their peers.

**Aim:**

The SOLID study aimed to investigate the feasibility of a definitive randomised controlled trial, comparing two behaviour change interventions to reduce risky substance use (illicit drugs and alcohol), and improve mental health, in young people in care.

**Methods:**

We recruited young people in care aged 12–20 years, self-reporting substance use within the previous 12 months and residing in 1 of 6 participating local authority sites in the North East of England. Participants were randomised to either i. Motivational Enhancement Therapy (MET), ii. Social Behaviour and Network Therapy (SBNT) or iii. Control (usual care). All interventions were delivered by trained drug and alcohol workers. Follow-up data were collected 12 months post recruitment. Feasibility for trial progression was compared to pre-specified stop: go criteria (recruitment of 60% of eligible participants, 80% of participants attending 60% of offered sessions and retention of 70% of participants at 12 month follow up).

**Results:**

Of 1450 eligible participants, 860 (59%) were screened for drug and alcohol use by social workers, 211 (24.5%) met inclusion criteria for the trial and 112 young people (7.7%) consented and were randomised. Sixty of these 112 participants (54%) completed 12-month follow-up questionnaires. Only 15 out of the 76 (20%) participants allocated to an intervention arm attended any of the offered MET or SBNT sessions.

**Conclusion:**

By reference to pre-specified stop: go criteria it is not feasible to conduct a definitive trial for SOLID in its current format. Despite co-designing procedures with staff and young people in care, the screening, referral and treatment pathway did not work here. Future work may require dedicated clinically embedded research resource to evaluate effectiveness of new interventions in services.

## Introduction

There were 78,150 young people in the care of the state in England on 31 March 2019, this represents 65 young people per 10,000 under the age of 18 years old [1]. Statistics show that there are nearly 48,000 young people in the care system in Australia [2] (termed ‘out-of-home’ care), and 269,690 in the US [3]. In the English context, looked after children are young people up to the age of 18 who are under the legal guardianship of local authorities [1]. Young adults who were in the care system, typically aged 18–21 years, are termed care leavers; these include young people who were previously under the legal care of local authorities and are still entitled to receive support [4]. This paper will use the term “young people in care” to describe both looked after children and care leavers.

Children and young people enter the care system for a multitude of reasons including abuse or neglect (61%), family ‘dysfunction’ (15%), family acute stress (8%), and absent parenting (7%) [4]. Many have been typically exposed to multiple adverse childhood experiences including parental poverty, absence of support networks, parental substance misuse, poor maternal and/or paternal mental health and early family disruption. These factors greatly increasing the risk of substance use, poor mental health, school failure and early parenthood within this population [5, 6].

Young people in care aged 11–19 years have a four-fold increased risk of drug and alcohol use compared to their peers [7]. A national survey of care leavers in England showed that 32% smoked cannabis daily [7] and data from 2012 showed that 11.3% of young people in care aged 16–19 years had a diagnosed substance use problem [8, 9]. In addition, young people in care have a significantly increased risk of mental health disorders and exposure to and/or involvement in risk taking behaviour [10]. Young people who engage in any one risk taking behaviour are more likely to engage in others [11, 12], with alcohol consumption, smoking and unprotected sexual intercourse often co-occurring [13, 14]. The transition to adulthood for young people in care can be difficult, resulting in social exclusion, exclusion from school and training, unemployment, health problems, homelessness and/or custody. If young people in care are to reach their full potential, effective and efficient interventions to reduce drug and alcohol use and support their mental health are essential. However, there is a paucity of evidence of effective interventions within this group.

The SOLID (SuppOrting Looked after children In Decreasing Drugs, and alcohol) pilot trial (RCT) aimed to test the feasibility of a definitive randomised controlled trial of two behaviour change interventions aimed at decreasing substance use (drugs and alcohol) of young people in care aged 12–20 years. We focused on interventions that could potentially impact substance misuse and mental health due to the profound impact these health issues have on the lifetime trajectories for young people in care and the fact that only 41% of young people in care referred to drug and alcohol service engage with treatment [15].

## Methods

### Formative work and process evaluation

Phase one of the SOLID study adapted two evidence-based behaviour change interventions, Motivational Enhancement Therapy (MET) and Social Behaviour and Network Therapy (SBNT), for use with young people in care. The MET and SBNT interventions modalities were chosen as they have both shown to be effective in decreasing substance use in adolescents [16–18]. The adaptation work and further rationale for choosing the MET and SBNT interventions has been previously published [19].

### Feasibility randomised controlled trial

SOLID was a three-arm feasibility randomised controlled trial; an outline of study procedures are presented in Table 1. A published trial protocol paper outlines all these procedures in detail [20].

**Table 1:** Study procedures.

Randomisation Unit	Individual
**Sample Size**	Minimum of 35 young people in care per arm (3 arm trial)
**Intervention arms**	active intervention arms:
	• 6 sessions of MET or
	• 6 sessions of SBNT
**Control arm**	Usual social worker delivered care with additional signposting to local drug and alcohol third sector services.
**Inclusion criteria**	• Young people in care aged ≥12 and ≤20 years.
	• Screened positive for being at risk of substance misuse i.e. score ≥1 on CRAFFT.
	• Provided informed consent to take part in the study: for young people under 16 years consent from foster parent/guardian (local authority) and assent from young person was required; for young people 16 years and over, consent was taken directly from young person.
**Exclusion criteria**	• Already in active treatment with drug and alcohol services.
	• Unable to access drug and alcohol services e.g. due to currently residing out of the study area, an imminent move out of area or being in young offender’s institution/prison/a secure unit.
	• Unable to give informed consent (due to acute or severe mental health difficulties, mental capacity or language barriers- this was assessed by the individual with parental responsibility).
**Outcome of feasibility trial**	Stop Go criteria:
	Definite Go (‘green light’) defined as:
	• ≥60% of eligible participants consenting to pilot trial
	• ≥80% of those in the intervention arms receiving intervention as planned
	• ≥ 70% retention of consented participants to 12 months follow up assessment
	Definite Stop (‘red light’) defined as:
	• <40% of eligible participants consenting to pilot trial
	• <20% of those in the intervention arms receiving intervention as planned
	• <50% retention of consented participants to 12 months follow up assessment.
**Primary Outcomes for definitive trial** (measured in pilot trial to assess feasibility of capture)	• Episodes of heavy episodic drinking (≥5 units in 1 day) in the preceeding 30 day period
	• Frequency of use of the most problematic classified substance in preceeding 30 days (data from TLFB-30)
**Secondary Outcomes for definitive trial (**measured in pilot trial to assess feasibility of capture)	• Mental health and wellbeing: Strength and Difficulties Questionnaire (SDQ) and the Warwick-Edinburgh Mental Well-being scale (WEMWBS).
	• Quality of life measured using the EQ-5D-5L.
	• Placement stability for the young person
	• Sexual behaviour: taken from the computer assisted self-interview (CASI) with questions relating to regret in sexual encounters and unprotected sex used in the ESPAD.
**Randomisation procedure**	Stratified randomisation based on placement type, local authority site and age band
**Blinding**	Blinding of group allocation was not possible for the young people in care participants, or for those delivering the intervention; however, the trial statistician and health economist were blinded to group allocation until the final analysis.

### Study registration

This study was registered with ISRCTN80786829.

### Ethics and consent to participate

A favourable ethical opinion was granted by Newcastle and North Tyneside 1 NRES Committee (16/NE/0123) on 22^nd^ April 2016.

### Setting

The study was conducted in six local authorities (Durham, Gateshead, Middlesbrough, Newcastle, Redcar and Stockton). The trial originally had four sites with two more being added to support the recruitment of young people in care into the study, further details can be seen in S1 File. The North East of England is an area of increased health and social care need and has the highest rates of poverty in the country with 24% of households living below the poverty line [21]. The region is, however, not uniform and encompasses a mixture of urban, peri-urban and semirural areas. The percentage of black and ethnic minority groups across the region also varies from 10% in Newcastle to 2% in Durham [22]. The North East region has 101 children in care per 10,000, far higher than the average rate for England of 65 per 10,000 [23]. Each area has a mixture of child placement types such as foster care placements, residential care homes and kinship carers. The area was chosen due to its high level of economic deprivation and young people in care.

### Screening and recruitment

The one-year recruitment period was 1^st^ November 2016 to 31^st^ October 2017. In-depth discussions were carried out within ‘looked after’ and leaving care teams in each of the local authority sites as part of trial protocol development. Training/briefing sessions were held in each site to encourage ‘buy-in’ from senior managers, team leaders and social workers. Social workers within the local authority study sites were requested to administer the CRAFFT (Car, relax, alone, forget, friends, trouble) screening tool to all young people aged 12–20 years, regardless of placement type, on their caseload. Individuals placed in young offenders’ institutes/prisons or secure units were excluded as they would be unable to attend intervention sessions.

For screening purposes, social workers verbally explained the study to young people under their care and provided a participant information leaflet. CRAFFT is a validated 9 item tool that has been used extensively with young people, and is sensitive and specific to identify problem substance use [24]. Young people self-completed the CRAFFT although social workers could provide support if requested. The screening questionnaire also contained simple information about the SOLID study and a contact detail form that young people were asked to complete if they were willing to be contacted as part of the ongoing study. If contact details were provided, they were forwarded to the research team who assessed eligibility to be recruited into the pilot trial. Amendments were made within the study to enable the research team to support social workers in completing the CRAFFT with young people in care. A £10 love to shop voucher (a voucher to be used in over 150 different shops) was introduced as an incentive for young people in care to complete the CRAFFT screening tool, further details can be seen in S1 File.

### Inclusion and exclusion criteria

The study eligibility criteria are summarised in Table 1. Slow early recruitment resulted in a protocol amendment such that young people reporting any substance misuse within the preceding 12 months, were deemed eligible (that is CRAFFT ≥1 rather than CRAFT ≥2); further details can be seen in S1 File.

### Study enrolment

Young persons in care who met inclusion criteria and who had registered an interest for the study were contacted by the research team. A convenient time and location were arranged directly with the young person and their carer (where appropriate) to take informed written consent for participation in the pilot trial. Most interviews took place within the young person’s place of residence. The research team talked through the participant information leaflets and consent form, ensuring potential trial participants had an opportunity to ask questions. For young people under 16 years, if the accompanying adult did not have parental responsibility (PR), the research team contacted the adult with PR to obtain informed consent. Written informed assent and/or consent was obtained prior to involvement in any part of the study, inclusive of baseline data collection as outlined in Table 1.

### Baseline and 12 months follow- up data collection methods

Baseline data collection took place at the first visit, post-consent and prior to randomisation. Follow up data collection occurred at 12 months post-recruitment. Baseline and follow-up questionnaires were self-completed via a tablet computer to facilitate greater privacy for participants. Questionnaires took approximately 20–30 minutes to complete. Table 2 provides information about the data collected at baseline and follow-up, along with details of proposed primary and secondary outcomes for a definitive trial if feasibility was demonstrated.

**Table 2:** Baseline and follow- up data collection.

Screening tool	Purpose	Scoring	Timepoint used
CRAFFT- (**C**ar, **R**elax, **A**lone, **F**orget, **F**amily or friends, **T**rouble) [24]	To reflect participants, own personal perspective of their drug and/or alcohol use and associated risky behaviours.	The screening questionnaire consists of 4 questions in part A and 6 questions in part B. All questions have an option of yes or no. Individuals received a score for each yes response they provided, the higher the score the more substances were being used and the more risky behaviours were taking place.	Screening
		For SOLID, young people screened positive for being at risk of substance misuse if they scored ≥1 on part A of CRAFFT.	
Alcohol Use Disorder Identification Test (AUDIT) [25]	To identify signs of hazardous and harmful drinking and identify mild dependence.	AUDIT is a 10-item scale. Scoring can range from 0–40, a score of 8+ is considered to indicate hazardous or harmful drinking. For the purpose of this study we use adult cut off points as a formal scoring system does not exist for children.	Baseline and Follow up
Alcohol, Smoking and Substance Involvement Screening Tool- Youth (ASSIST-Y) [26, 27]	To identify moderate and high-risk scores broken down by age and substance.	Questions relate to 9 different substances. **10 to 14 years of age**	Baseline and follow up
		For tobacco, alcohol, inhalants–score 2 to 5 moderate risk, score of > 6 high risk. Scores >2 in any other substance indicates high risk	
		**15 to 17 years of age**	
		Any injection of drugs is high risk.	
		For tobacco and cannabis—score 2 to 11 moderate risk. For alcohol score 5 to 17 moderate risk. For cocaine, sedatives, opioids, NPS and ‘other’ drugs score 2 to 6 moderate risk. For amphetamines, inhalants and hallucinogens score 2 to 8 moderate risk.	
		High risk scores tobacco and cannabis (>12), alcohol (>18), cocaine, sedatives, opioids, NPS and ‘other’ (>7) and amphetamines, inhalants and hallucinogens (> 9).	
Strengths and Difficulties Questionnaire (SDQ) [28, 29]	To measure self-reported mental health and wellbeing.	25 questions to assess four difficulty subscales and measure pro-social behaviour. Total difficulties scores range from 0–40, each one-point increase in this score corresponds with an increased risk of developing mental health disorders; close to average (0–14), slightly raised (15–17), high (18–19), very high (20–40).	Baseline and Follow up
Warwick-Edinburgh Mental Wellbeing Scale (WEMWBS) [30].	To measure subjective well-being and psychological functioning.	14 item scale with each item scored 1 (none of the time) to 5 (all of the time) on a Likert scale. Total scores range from 0–70, higher scores denote positive mental health; very low score (0–32), below average (32–40), average score (40–59), above average score (59–70).	Baseline and Follow up
EQ-5D-5L [31, 32]*	To assess health-related quality of life- five dimensions of health-related quality of life are assessed (mobility, self-care, usual activities, pain/discomfort and anxiety/depression).	Each dimension has five potential responses ranging from 1 (no problems) to 5 (extreme problems).	Baseline and Follow up
		The digits for the five dimensions can be combined into a 5-digit number that describes the patient’s health state E.g. 1,1,1,1,1- no problems in any domain or 5,5,5,5,5- extreme problems in all domains.	
		Once completed each individual would have a 5-digit code for EQ 5D 5L, such as 41325.	
	In addition, a vertical visual analogue scale (VAS) is used for participants to self-rate their health.		
		VAS scores range from 0- ‘the worst health you can imagine’ to 100- ‘the best health you can imagine’.	
Time Line Follow Back- 30 (TLFB- 30) [33]	The TLFB is a drinking assessment method that obtains estimates of daily drinking (e.g., pattern, variability, and magnitude of drinking). over a 30-day period.	The TLFB in SOLID sought to identify heavy episodic drinking (high intensity ‘binge’ drinking is defined as the 'number of occasions where 5 or more standard drink units are consumed on a single drinking day' as used in the ESPAD survey. [34] This measure was chosen as an objective measure of likely intoxication or ‘drunkenness’ which in turn is associated with behavioural risk taking.	Follow up

* The youth and adult version of the EQ-5D-5L tool can be used for 12–15 year olds and for 16 years plus the adult version is recommended, therefore we used the adult version of the EQ-5D-5L with all participants for consistency across the dataset.

### Randomisation and allocation

The trial administrator used a secure online system to conduct the randomisation. Individual randomisation was stratified by placement type (residential/non-residential), local authority site and age band (12-14/over 14), to reflect risk profile for substance use. Blinding of group allocation was not possible for the young people in care participants, or for those delivering the intervention; however, the trial statistician and health economist were blinded to group allocation until the final analysis.

Participants received a letter informing them of their allocated treatment group following randomisation and what they could expect to happen next. Participants allocated to an intervention arm were contacted via telephone by a drug and alcohol worker from their local young people’s drug and alcohol service who introduced themselves and arranged a convenient time and place to meet for an initial meeting. Participants allocated to ‘usual care’, were still eligible to receive support for substance misuse; this required their allocated social worker to make a referral to drug and alcohol services using the standard care pathway.

### Study interventions

The MET and SBNT interventions offered within SOLID differed from standard care as shown in Table 3. The drug and alcohol practitioners were trained to deliver the MET or SBNT interventions using a treatment manual in a standardised way. The usual care offer could be inclusive of multiple different approaches and was not standardised.

**Table 3:** Comparisons between SOLID interventions and usual care.

SOLID Interventions	Standard Care (2017/2018)
• Young people were offered SBNT or MET within 6 weeks of randomisation.	• 97% of young people accessing specialist treatment had to wait 3 weeks or under to start an intervention.
• Young people were ineligible if they were already in active treatment	
	• Interventions not standardised and used several theoretical frameworks
• Interventions would last a maximum of 6 sessions delivered over 12 weeks	
	• Young people can access 1 or more interventions at the same time
	• Average (mean) treatment episode was 4.9 months.

MET is a directive, client-centred counselling approach. The basic assumption is that it is the therapist’s role to create an environment to enable change but that the motivation and responsibility for change lie within the client [35].

SBNT utilises cognitive and behavioural strategies as part of a systematic counselling approach to help identify and build a positive social network to support behaviour change in relation to goal attainment regarding problem substance use [36].

Both interventions comprised a maximum of six, 1- hour sessions offered weekly or fortnightly over a maximum of 12 weeks. Interventions were offered at a location convenient to the participant. Further details regarding the interventions and the rationale for choosing them are reported elsewhere [19].

Acceptability data collected within the process evaluation included qualitative data from 109 stakeholders (37 children in care and 72 professionals). An additional paper presenting the findings from the qualitative process evaluation, including acceptability of the intervention to the recipients (young people in care) and deliverers (drug and alcohol workers) is in progress.

### Statistical analysis

As this was a pilot trial, the primary outcomes focused on feasibility, reporting the number of eligible participants seen over the recruitment period and the resulting rates of recruitment, compliance with randomisation and data completion. The main statistical analyses were descriptive and aimed at informing the design, sample size and conduct of a future definitive trial. No formal statistical comparisons were made since the trial was not powered to detect differences in participant-reported outcomes. As outlined by Teare et al 2014 on sample size requirements to estimate key design parameters from external pilot randomised controlled trials, the target sample size was determined as a minimum of 35 respondents in each trial arm at 12-month follow-up. This is reported to be sufficient to estimate the critical parameters of a continuous primary outcome (in our case heavy episodic drinking (high intensity ‘binge’ drinking) in the preceding 30 day period derived from the TLFB/30) to the necessary degree of precision for the definitive trial. [37] Assuming a 30% loss to follow-up in line with previous research with young people in care [38] the required sample size was 46 per arm to be recruited which we rounded up to 50 [20].

### Health economic analysis

The economic analysis aimed to assess the feasibility of collecting cost and benefit data for a within-trial economic analysis as part of a definitive trial. A value of information analysis (VOI) [39] was proposed, incorporating data from the literature and the feasibility study. The data needed for the VOI analysis includes cost data for the intervention and outcome data measured by QALYs, derived from EQ-5D responses. The purpose of the proposed value of information (VOI) analysis would have been to help clarify the economic case for a definitive study and, by using a variant of VOI analysis, utilise expected value of sampling of information to inform decisions on the optimal sample size for a definitive trial. We collected data on the resources needed to deliver the study including all intervention activity and study measurement tool completion rates.

### Stop-go criteria

In keeping with the ADePT framework [40], we used stop: go criteria on participant recruitment rate, adherence with the intervention (treatment fidelity), and retention at 12 month follow-up to assess feasibility of the pilot trial. We expanded our criteria to include, intervention acceptability. The progression criteria to move from pilot to full trial were divided into three categories (green, red and amber) as outlined by Bugge 2013 [40] and assessed in Table 7 of the results section.

## Results

### Screening–population risk profile

Of 1450 young people in care aged 12–20 years in six participating local authority sites, 860 (59%) self-completed and returned the CRAFFT screening tool. Of these individuals, 369 (43%) reported using one or more substance (drugs and/or alcohol) in the preceding 12 months. Frequency of reported substance use was not collected as the CRAFFT is a short screening questionnaire.

Alcohol was the most commonly reported substance, used by 347 (40%) of young people in care, followed by 166 (19%) reporting cannabis use; 43 (5%) other substances including prescription and over the counter drugs and 39 (4.5%) use of ‘legal highs’. In total, 161 (18.7%) young people in care had used more than one substance in the last 12 months with 54 (6.3%) reporting use of three or more substances.

Of 278 (32%) individuals who screened positively (≥1) on the CRAFFT tool: 97 (11%) reported that they had been in a **C**ar driven by someone (including themselves) who was under the influence of alcohol or drugs; 153 (18%) used alcohol or drugs to **R**elax, feel better about themselves or fit in; 128 (15%) used alcohol or drugs whilst they were **A**lone; 148 (17%) had **F**orgotten things they did whilst under the influence of alcohol or drugs; 119 (14%) stated that **F**amily or friends had told them they should cut down on the drinking or drug use; and 130 (15%) had got into **T**rouble due to their alcohol or drug use. Just over two thirds (n = 582, 68%) of the young people reported that they had not engaged in risky behaviour, the rest (n = 278; 32%) reported one or more risk taking behaviours associated with substance use on CRAFFT. Young people could report up to six risky behaviours, (n = 32, 4%) reported 5 risky behaviours and (n = 17, 2%) reported engaging in all six risky behaviours.

Screening across the sites was slow and took 12 months to complete. Bespoke support mechanisms were put in place to facilitate screening, such as researchers being embedded in four study sites and one site using a ‘stop the clock’ technique to provide social workers with dedicated time to contact young people on their caseload.

### Recruitment

Of the 860 young people in care who returned the screening forms, 356 (41%) did not provide consent to be contacted, while a further 269 consented to be contacted but were ineligible as they reported no substance use in the previous 12 months.

Of 235 young people who consented to be contacted and were eligible in terms of reporting substance use; 24 were subsequently excluded as follows: 11 were already engaged with drug and alcohol services; six had an imminent move out of area; three had ‘aged’ out of care and one each was excluded due to—recall to young offender’s institution, mental health hospitalisation, a learning disability and turned 21 since completing the CRAFFT screening.

In addition, 24 young people provided consent to contact and were potentially eligible participants, however they were then excluded as they did not provide any contact details and 75 declined to take part for the following reasons, 18 did not feel they had a substance use problem, 11 were uninterested in the study; three felt their drinking was legal; 34 declined without providing a specific reason; and 8 were involved with criminal justice or mental health services.

**Fig 1:**
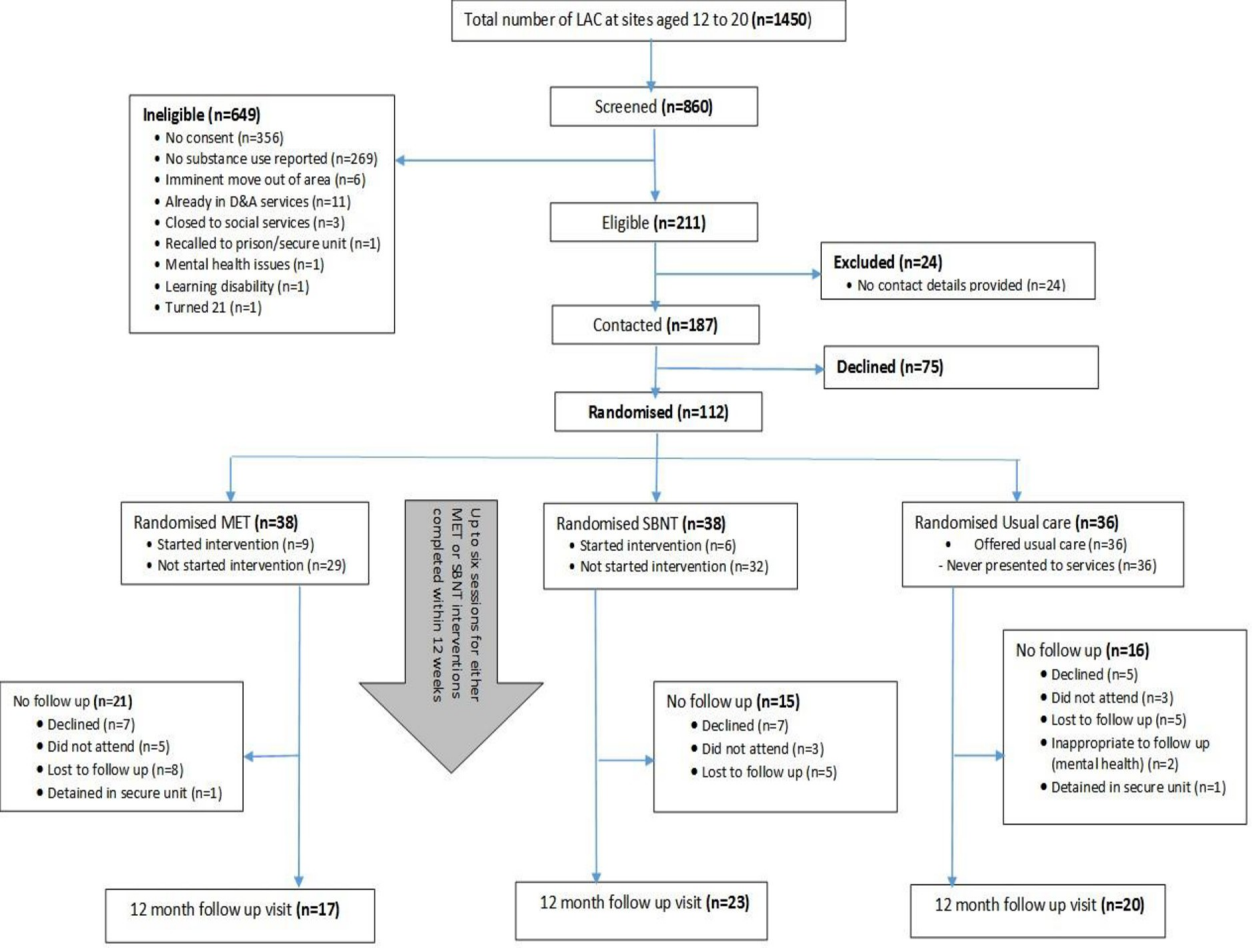
Consort diagram.

Consequently, 112 individuals out of the 211 (53%) who met the inclusion criteria for the trial were enrolled into the study; representing 13% of those returning a screening questionnaire. Fig 1 presents the study Consort diagram, and identifies the numbers screened, enrolled into the study, allocated to each intervention arm and contacted at 12-month follow up.

As per original protocol, we aimed to recruit 150 young people [20]. In total, we recruited 112 participants representing 75% of our original recruitment target. We retained 60 participants (54%) at 12-month post recruitment, which was a 46% loss at follow up.

### Baseline participant characteristics

The distribution of demographic variables was broadly similar across the trial arms (Table 4), although the control group seemed to contain disproportionately more females. There were very few non-white participants in all arms, this reflects the ethnic mix across the local authorities as the population is 91% white [41–43].

**Table 4:** RCT Baseline characteristics.

	MET (n = 38)	SBNT (n = 38)	Usual care (n = 36)	Overall (n = 112)	Screen (n = 860)
**Gender**					
Male	17 (45%)	21 (55%)	12 (33%)	50 (45%)	440 (51%)
Female	21 (55%)	17 (45%)	24 (67%)	62 (55%)	409 (48%)
missing					11 (1%)
**Age (years)**					n = 858
Median (LQ, UQ)	18 (16, 19)	17 (16, 18)	18 (16, 19)	17 (16, 19)	16 (14, 18)
Range (min, max)	(13, 21*)	(13, 20)	(13, 20)	(13, 21*)	(12, 20)
Mean (SD)	17.5 (2.1)	17.0 (1.9)	17.3 (2.0)	17.3 (2.0)	16.1 (2.4)
missing					2
**What do you do during the day?**					Not available at screening
In school	7 (18%)	10 (26%)	7 (19%)	24 (21%)	
Under 16 not in school	2 (5%)	1 (3%)	0 (0%)	3 (3%)	
6^th^ form/college/ university	8 (21%)	12 (32%)	8 (22%)	28 (25%)	
In training /apprenticeship	4 (11%)	4 (11%)	1 (3%)	9 (8%)	
16+ not in training, employment or	15 (39%)	8 (21%)	17 (47%)	40 (36%)	
education	2 (5%)	3 (8%)	2 (6%)	7 (6%)	
Over 16 and employed	0 (0%)	0 (0%)	1 (3%)	1 (<1%)	
**Placement type**					
Foster Care	9 (24%)	12 (31%)	13 (36%)	34 (30%)	452 (52%)
Residential home	8 (21%)	6 (16%)	6 (17%)	20 (18%)	83 (10%)
Own accommodation	21 (55%)	17 (45%)	11 (31%)	49 (44%)	
With parents	0 (0%)	1 (3%)	5 (14%)	6 (5%)	
other	0 (0%)	2 (5%)	1 (3%)	3 (3%)	249 (29%)
missing					76 (9%)
**Site**					
Newcastle	6 (16%)	7 (18%)	7 (19%)	20 (18%)	189 (22%)
Durham	12 (32%)	10 (26%)	11 (31%)	33 (29%)	201 (23%)
Gateshead	4 (11%)	4 (11%)	4 (11%)	12 (11%)	125 (15%)
Middlesbrough	6 (16%)	6 (16%)	5 (14%)	17 (15%)	146 (17%)
Redcar	2 (5%)	3 (8%)	2 (6%)	7 (6%)	95 (11%)
Stockton	8 (21%)	8 (21%)	7 (19%)	23 (21%)	104 (12%)
**Ethnic group**					Not available at screening
White British	38 (100%)	34 (89%)	34 (94%)	106 (95%)	
Other ethnicity	0 (0%)	4 (11%)	1 (3%)	5 (4%)	
Missing	0 (0%)	0 (0%)	1 (3%)	1 (<1%)	

### Summary of data collected at baseline

Table 5 provides a summary of data collected at baseline regarding alcohol use and mental health. Given the use of the tablet computer for data collection, if the young people attended the meeting, data were successfully collected on all scales. Respondents were required to provide an answer for one question before being able to move on to the next question, thus there was no missing data for participants who agreed to fill in the questionnaire at either baseline or follow up. There was no indication that any scale led to a lower response rate, therefore they appeared to be acceptable.

**Table 5:** Summary of baseline alcohol and mental health data by trial arm and combined across arms.

Variable	MET	SBNT	Usual Care	Overall
	Baseline (n = 38)	Baseline (n = 38)	Baseline (n = 36)	Baseline (n = 111) **
**AUDIT**				
Hazardous alcohol	24 (63%)	17 (45%)	20 (57%)	61 (55%)
**ASSIST- Y**				
Alcohol	37 (97%)	37 (97%)	34 (94%)	108 (97%)
Tobacco	30 (79%)	30 (79%)	33 (92%)	93 (84%)
Cannabis	30 (79%)	21 (55%)	30 (83%)	81 (73%)
Cocaine	14 (37%)	12 (31.5%)	14 (39%)	40 (36%)
Amphetamine	11 (29%)	11 (29%)	5 (14%)	27 (24%)
Sedative	10 (26%)	7 (18%)	10 (28%)	27 (24%)
Hallucinogens	7 (18%)	6 (16%)	6 (17%)	19 (17%)
Novel Psychoactive Substance (NPS)	9 (24%)	7 (18%)	3 (8%)	19 (17%)
Opioid	5 (13%)	3 (8%)	3 (8%)	11 (10%)
Inhalants	2 (5%)	3 (8%)	4 (11%)	9 (8%)
Other	2 (5%)	0 (0%)	1 (3%)	3 (2.7%)
**Warwick-Edinburgh Mental Well-Being Scale (WEMWBS)**				
Very low score (0–32)	8 (21%)	3(8%)	3 (9%)	14 (13%)
Below average score (32–40)	9 (24%)	10 (26%)	9 (26%)	28 (25%)
Average score (40–59)	15 (39%)	20 (53%)	22 (63%)	57 (51%)
Above average score (59–70)	6 (16%)	5 (13%)	1 (3%)	12 (11%)
**Strength and Difficulties Questionnaire (SDQ)**				
Close to average score (0–14)	15 (39%)	22 (58%)	15 (43%)	52 (47%)
Slightly raised score (15–17)	7 (18%)	6 (16%)	3 (9%)	16 (14%)
High score (18–19)	7 (18%)	3 (8%)	4 (11%)	14 (13%)
Very high score (20–40)	9 (24%)	7 (18%)	13 (37%)	29 (26%)
**EQ- 5D-5L- Anxiety and Depression**				
No problems	15 (39%)	16 (42%)	17 (48.5%)	48 (43.2%)
Slight problems	7 (18%)	10 (26%)	10 (28.5%)	27 (24.3%)
Moderate problems	5 (37%)	7 (18%)	5 (14%)	17 (15.3%)
Severe problems	7 (18%)	3 (8%)	2 (6%)	12 (10.8%)
Extreme problems	4 (29%)	2 (5%)	1 (3%)	7 (6.3%)
**EQ-5D-5l VAS**				
mean score	70.44737	72.60526	71.22857	71.43243
Range (min-max)	(5–100)	(10–100)	(20–100)	(5–100)

** Note that participant 36 (female, 19 years) was randomised but their baseline data did not transfer electronically to the trial database due to a technical fault. This participant’s data could not be included in the baseline analysis, leaving 111 participants providing baseline data.

### Alcohol and substance use

At baseline, 61 (55%) of participants were defined as a ‘hazardous or harmful’ drinker as identified by the AUDIT tool [44]. Indeed, alcohol was the most commonly used substance (reported by 97% of respondents at baseline) followed by tobacco and cannabis.

### Mental health and wellbeing

Fourteen (13%) participants reported a ‘very low’ mental health and well-being, and a further 28 (25%) below average scores. In total, 29 (26%) of participants reported a ‘very high’ total difficulties score, indicating an increased risk of developing a mental health disorder.

### Health related quality of life

Around 17% of participants reported sever or extreme problems regarding anxiety or depression. The overall visual analogue scale (VAS) score showed high variability with scores ranging from 5–100, with a mean score of 71. 43.

### Delivery of intervention and intervention attendance

Just 15 out of 76 (20%) participants allocated to an intervention group attended at least one session of the intervention (across MET and SBNT arms combined). In the MET arm, 5 of the 38 (13%) randomised participants took up at least one session, three young people attended two sessions and one young person attended all six sessions. Similarly, in the SBNT arm, 4 of the 38 (10%) randomised participants attended at least one session, one young person attended two sessions and one attended three sessions.

### Fidelity of the interventions

Overall, 15 young people engaged with a MET/SBNT intervention and a fully completed practitioner log was returned. Twelve out of the 15 (80%) young people who attended any sessions were recruited under the original screening threshold of reporting ≥2 risky behaviours, compared to only 3 recruited under the reduced criterion of any substance use in the last 12 months. In total, 26 intervention delivery sessions were delivered; MET (n = 17) and SBNT (n = 9) and of these, practitioners only completed 9 recordings; MET (n = 3) and SBNT (n = 6). Reasons provided for not recording sessions included participant refusal (n = 8) and the practitioner ‘forgetting’ to turn the recorder on and professionals not feeling comfortable with their practice being audio recorded (n = 9). The returned log number was too small to conclude whether the tool is acceptable to use in a definitive trial. We used Carroll et al’s definition of fidelity to assess whether MET/SBNT sessions were delivered as planned in terms of content, frequency, duration and coverage [45]. We assessed the quality of the intervention delivery (treatment fidelity) by applying the validated process rating scale (PRS) developed within the UKATT trial [46] further details provided in S2 File. Unfortunately, the small number of audio recordings and fully completed practitioner logs meant that we could not accurately assess the fidelity of the interventions being delivered. The anxiety of both participants (n = 8) and practitioners (n = 9) regarding the audio recordings suggests that an alternative method of assessing the fidelity of intervention delivery would need to be considered in a definitive trial.

### 12 months follow up data

Of 112 enrolled participants, sixty (54%) completed follow up at 12 months post recruitment, nineteen (17%) declined follow up, eighteen (16%) were uncontactable, eleven (10%) arranged follow up meetings but then did not attend, two were detained in a secure unit and two were deemed inappropriate to follow up by their allocated social worker. When attempting follow-up, the contact details originally provided were no longer current; 58 (52%) of participants, had changed their contact number and, due to placement instability, 54 (48%) of participants had changed address and 40 (36%) had changed both their contact number and address. A summary of follow up data at 12 months are provided for descriptive purposes only, as shown in Table 6.

**Table 6:** 12-month descriptive data.

Variable	MET	SBNT	Usual Care	Overall
	12 months (n = 17)	12 months (n = 23)	12 months (n = 20)	12 months (n = 60)
**AUDIT**				
Hazardous alcohol	12 (71%)	7 (30%)	10 (50%)	29 (48%)
**ASSIST- Y**				
Alcohol	17 (100%)	23 (100%)	20 (100%)	60 (100%)
Tobacco	14 (82%)	18 (78%)	18 (90%)	50 (83%)
Cannabis	12 (70.5%)	14 (61%)	14 (70%)	40 (67%)
Cocaine	8 (47%)	5 (22%)	7 (35%)	20 (33%)
Amphetamine	7 (41%)	7 (30%)	3 (15%)	17 (28%)
Sedative	6 (35%)	4 (17%)	5 (25%)	15 (25%)
Hallucinogens	4 (23.5%)	3 (13%)	4(20%)	11 (18%)
Novel Psychoactive Substance (NPS)	3 (18%)	3 (13%)	2 (10%)	8 (13%)
Opioid	2 (12%)	1 (4%)	1 (5%)	4 (7%)
Inhalants	2 (12%)	1 (4%)	2 (10%)	5 (8%)
Other	0 (0%)	0(0%)	0 (0%)	0 (0%)
**Warwick-Edinburgh Mental Well-Being Scale (WEMWBS)**				
Very low score (0–32)	5 (29%)	4 (17%)	3 (15%)	12 (20%)
Below average score (32–40)	2 (12%)	2 (9%)	2 (10%)	6 (10%)
Average score (40–59)	9 (53%)	15 (65%)	14 (70%)	38 (63%)
Above average score (59–70)	1 (6%)	2 (9%)	1 (5%)	4 (7%)
**Strength and Difficulties Questionnaire (SDQ)**				
Close to average score (0–14)	5 (29%)	13 (57%)	12 (60%)	30 (50%)
Slightly raised score (15–17)	1 (6%)	4 (17%)	2 (10%)	7 (12%)
High score (18–19)	3 (18%)	2 (9%)	2 (10%)	7 (12%)
Very high score (20–40)	8 (47%)	4 (18%)	4 (20%)	16 (27%)
**EQ- 5D-5L- Anxiety and Depression**				
No problems	2 (12%)	10 (43%)	8 (40%)	20 (33%)
Slight problems	5 (29%)	6 (26%)	5 (25%)	16 (27%)
Moderate problems	6 (35%)	4 (17%)	5 (25%)	15 (25%)
Severe problems	3 (18%)	2 (7%)	1 (5%)	6 (10%)
Extreme problems	1 (6%)	1 (4%)	1 (5%)	3 (5%)
**EQ-5D-5l VAS**				
mean score	65.47059	80.47826	69.5	72.56667
Range (min-max)	(40–100)	(35–100)	(10–100)	(10–100)
**TLFB: Episodes of heavy drinking (≥5 units in 1 day) in the preceding 30-day period**				
Number at follow up (% of randomised)	17 (45%)	22* (58%)	20 (56%)	59 (53%)
Median (LQ, UQ)	1 (0, 4)	0 (0, 2)	1.5 (0, 5.5)	1 (0, 4)
Range (min, max)	(0, 10)	(0, 7)	(0, 9)	(0, 10)

*Note that one participant did not complete the TLFB at follow up. They did however complete all other questionnaires at that time

### Stop: Go criteria

All the assessment criteria set against our stop: go criteria were judged to be amber or red indicating a definitive trial was not likely to be feasible, see Table 7.

**Table 7:** Assessment of the SOLID stop/go criteria.

	Green criteria	Amber criteria	Red criteria	Outcome
% Eligible young people in care consenting to trial	≥60%	40–60%	<40%	**53%**- Amber
% of participants randomised to intervention attending 60% of offered sessions	≥80%	20–80%	<20%	9%-Red
% of randomised participants retained to 12 months follow-up	≥70%	50–70%	<50%	**54%-** Amber
Intervention delivered with fidelity	Yes (scored 3–4 using PRS**scale)	Unclear (scored 1–2 using PRS scale or insufficient data to score)	No (scored 0 using PRS scale)	**Unclear**- Amber
Intervention perceived acceptable by young people in care and workers.	Yes	Unclear	No	**Unclear**- Low uptake of intervention by children, insufficient data to score—Amber
Value of information analysis shows future research is worthwhile	Worthwhile	Unclear	Not worthwhile	Noavailabledata-Red

**Process rating Scale- A validated process rating scale which was developed in the UKATT trial [46] was used, the scale covers both MET and SBNT, see S2 File.

As Table 7 identifies, the criteria to determine the feasibility of progressing to a full definitive trial were not met. The combination of multiple steps in the study process and the time lost between screening and first appointment set up within the current referral pathways, meant that the process was not swift enough to engage participants in the trial and the interventions.

### Health economic analysis

Unfortunately, no useable data were available from the feasibility study and insufficient data were available from the literature to conduct the economic evaluation modelling exercise that underpins a value of information analysis. Exploratory return on investment analysis was conducted to identify the range of values for benefits and total costs consistent with a return of investment of a minimum level, this is available upon request to the authors.

## Discussion

Young people in care are an under-researched group and there are well-known challenges in conducting trials in this group, including the lack of research infrastructure in Social Care [47]. We undertook extensive formative work, adapted interventions with young people in care and relevant staff [19] and then conducted a pilot trial and parallel process evaluation. Despite the detailed preparatory work, we found that is it not feasible to conduct a trial of the adapted substance use behaviour change interventions within the current screening, referral and treatment pathway. Just 860/1450 (59%) of eligible young people in care across 6 local authorities were screened and only 13% subsequently enrolled. Of those enrolled in the study, very few took up the MET or SBNT interventions, making fidelity assessment impossible. All these parameters indicate that a definitive trial is not achievable at the current time. SOLID was funded as a feasibility trial only. It met its aim to assess the feasibility of a definitive trial; as feasibility was not proven, no further funding was sought.

Local authorities undertaking screening reported having multiple competing demands, inclusive of significant periods of time without senior managers in post and OFSTED (Office for Standards in Education, Children’s Services and Skills) inspections. [OFSTED provides independent inspections to ensure that organisations providing care services do so to a high standard [48]]. The unforeseen complexities influenced progress and major challenges were found in both screening and recruitment of young people in care into the study. Overall, recruiting from local authority sites was highly time- and resource-intensive, to the point that we must conclude that mass screening of young people in care for trial eligibility is not feasible as part of ‘standard social work practice’. Given the difficulties experienced, an alternative method of screening and recruitment which is not dependent upon social workers as gatekeepers would be required in any future study.

An embedded researcher within social care departments could facilitate screening of young people in care and engagement of social workers in study procedures. A researcher within this role has the potential to be jointly managed by local authorities and universities, facilitating clearance to engage clients. Within our current study, we did not provide any training regarding research methods to social workers. However, we did carry out several engagement sessions with staff. In hindsight training in research methods could have had the potential to improve study outcomes and organisational readiness to engage in the research process. Cheetham et al. (2018) [49] have provided an example of establishing an embedded researcher within a local authority environment, which led to an increased understanding of conducting public health interventions within the organisation. Within a future study, an embedded researcher, or indeed a trained local authority member of staff seconded to a research role, could provide a stable point of contact within the complex local authority setting to facilitate more successful screening, recruitment and retention into a trial.

Twelve out of the 15 (80%) young people who attended any sessions were recruited under the original screening threshold of reporting ≥2 risky behaviours, compared to only 3 recruited under the reduced criterion of any substance use in the last 12 months. This would suggest that individuals reporting higher levels of risky substance use behaviour might be more likely to attend intervention sessions. We had widened screening criteria to encourage recruitment into our trial, but this might have come at the cost of salience for participants.

Though 161 (18.7%) participants reported poly-substance use on the CRAFFT, the majority did not perceive their alcohol and/or drug use as problematic. Linked formative work suggested that young people in care feel that other challenges cause them more problems than substance use per se [19, 50]. Currently, drug and alcohol interventions are delivered in a vertical manner by a specialist service. A shift to more horizontal intervention delivery in which several interrelated issues can be tackled at once might be more beneficial [51, 52]. This could be accommodated by implementing a more person-centred (place- based) approach to meet the needs of young people in care [53]. This approach advocates that services (in this case social work and drug and alcohol counselling) develop a more integrated delivery system and work together to improve health and care outcomes based on the needs of the population they serve. In our sample, many young people felt that substance use was a symptom rather than a cause of problems (42). Consequently work on any substance use needs to be set into a wider context of adverse childhood experiences [54] and difficulties experienced in care [37] inclusive of mental health difficulties.

When completing the EQ-5D-5L, 19 (17%) of respondents reported suffering from severe or extreme anxiety and depression. This was mirrored by the high rates of distress in our baseline SDQ measures. Future work with young people in care may consider a measure of quality of life related to mental health [55]. In addition, the EQ-5D has ceiling effects which another utility tool such as the SF-6D does not (although that tool has floor effects), these alternative tools should be considered in future work.

### Limitations

Our study took place during a period of continued austerity and organisational change, resulting in social workers experiencing constraints in time and resources to conduct the additional task of screening and recruiting young people in care into the SOLID study.

### The delivery of future interventions

The MET and SBNT interventions were chosen as they have both shown to be effective in decreasing substance use in adolescents [16–18]. There is strong evidence for the use of brief advice and counselling for heavy drinking (akin to more frequent substance used in this sample), with little indication that longer counselling approaches provide any additional benefit [56]. However, most of this literature relates to adults and older adolescents from the general population. It is clear from this study that young people in care have higher substance use risk profiles compared to the general population. Therefore, they may need more specifically tailored support due to the antecedents and consequences of being in the statutory care system.

A broader tiered approach, such as the thrive model currently used by Child and Adolescent Mental Health Services (CAMHS), may be needed to help accommodate the view that experience of being in care itself can cause distress, which is a significant risk factor for multiple risk-taking behaviours and poor mental health. The thrive model promotes five categories; thriving, getting advice and signposting, getting help, getting more help and getting risk support [57]. Thus, the category of ‘getting risk support’ could incorporate the delivery of substance misuse counselling and/or intervention delivery and could be part of a process that understands the wider determinants of risk and associated risk behaviour. By delivering the adapted intervention according to the thrive categories, it would allow a person centred and needs-led approach to be delivered to young people in care regarding their substance use, which could accommodate both a preventative/harm reduction intervention or more intensive therapeutic support to be delivered as required.

This study tried to deliver novel interventions using existing alcohol and drug referral systems and pathways. We have highlighted challenges in recruiting and retaining individuals and also that young people in care did not take up the intervention offered. Findings within our formative work, highlighted how young people in care find it difficult to establish relationships and trust other people [19, 51]. On reflection, our process of referring participants to trained drug and alcohol staff (which fits current service provision) may have contributed to losing participants within the study as they had to move through multiple steps before being offered interventions by a specialist worker. A new, more responsive way of working to deliver these interventions is needed. One possible solution to facilitate a smoother transition might be to assign a drug and alcohol worker to be co-located within residential units and social care teams to help provide more integrated care [53]. This would have the benefit of having the skills of a specialist worker embedded into existing care teams, enabling a more responsive approach to the complex needs of young people in care. Future studies would need to consider the best way to engage young people in care in interventions that aim to prevent future harms to them, and this may require additional resources.

## Conclusion

Young people in care are significantly more likely than peers in the general population to use substances and have severe mental health morbidity. Despite this recognised need, a definitive trial of adapted behaviour change interventions to help reduce substance use was demonstrated not to be feasible. The screening, referral and treatment model used in SOLID was problematic; future interventions may need to be delivered more opportunistically with fewer steps in the process. However, given the wider risks inherent in being placed in the care system, we may need to embed substance use work within a context of understanding wider determinants of risk behaviour.

## Supporting information

S1 File (DOCX)

S2 File (DOCX)

S3 File (DOCX)

S1 Checklist (DOC)

## Abbreviations

ASSIST-Y: Alcohol, Smoking and Substance Involvement Screening Test- Youth
AUDIT: Alcohol Use Disorders Identification Test
CAMHS: Child and Adolescent Mental Health Services
CRAFFT: Car, Relax, Alone, Forget, Friends, Trouble(s)
MET: Motivational Enhancement Therapy
NEET: Not in education, employment or training
OFSTED: Office for Standards in Education, Children’s Services and Skills
PR: Parental responsibility
RCT: Randomised control trial
SBNT: Social Behaviour and Network Therapy
SDQ: Strengths and Difficulties Questionnaire
SOLID: Supporting Looked After Children and Care Leavers In Decreasing Drugs, and Alcohol)
UK: United Kingdom
UKATT PRS: United Kingdom Alcohol Treatment Trial Process Rating Scale
WEMWBS: Warwick-Edinburgh Mental well-being scale
VAS: Visual Analogue Scale
