# Supplementary material for: A pilot feasibility randomised controlled trial of two behaviour change interventions compared to usual care to reduce substance misuse in looked after children and care leavers aged 12-20 years: The SOLID study
Source: PLoS One. 2020 Sep 8;15(9):e0238286. doi: 10.1371/journal.pone.0238286 (PMC7478815; doi:10.1371/journal.pone.0238286)
Supplement: S1 File — (DOCX) [file pone.0238286.s001.docx]

**S1: Supporting information file- RCT Amendment History**

## Amendment History

| **Amendment Number** | **Protocol version no.** | **Date issued** | **Author(s) of changes** | **Details of changes made** |
| --- | --- | --- | --- | --- |
| SA1 | 2.0 | 05.04.2017 | Dr Raghu Lingam | **Amendment 1: Introduction of an incentive to complete CRAFFT screening tool**  The Looked After Children and care leavers involved in the study will receive a £10 voucher as a thank you for taking time to complete the CRAFFT screening tool. This amendment is proposed due to direct feedback from LAC service managers and social workers, whom felt that YP would appreciate an acknowledgement of their support and time within the study. Young people will receive the £10 voucher upon completion of the screening tool, regardless of whether they provide contact details to take part in the RCT element of the study.  The CRAFFT screening tool is remaining the same and it will still be completed by a social worker as originally presented, the only alteration is that young people will receive a £10 voucher upon completion of the screening form, this was not in the original application.  **Amendment 2: Researchers to support social workers to complete CRAFFT screening tool with LAC**  Social workers within the Local Authority sites have report to the research team that they are finding it problematic completing the CRAFFT screening tool with young people on their caseload due to time constraints and the current pressures on local authority staffing levels. Social work teams have identified that the research team could support the social workers by researchers liaising directly with keyworkers in the residential children’s home. Therefore, it is proposed that researchers could receive a list of the children’s homes and the managers names (nothing identifiable to the young person due to confidentiality). The research will arrange an appropriate time to visit each home and introduce the study to the residential key workers and the young people. The residential key workers would act as a gatekeeper and approach the young people to request that they complete the screening tool. The screening tool could be completed whilst the researcher was present in case participants require any additional support or assistance.  **Amendment 3: Introduction of additional Sites**  We have approached, Gateshead and Sunderland Local Authorities to become additional sites for SOLID. The original application mentioned Teesside as a recruitment area, for clarity this entailed recruitment from Middlesbrough, Stockton, Redcar and Cleveland. This amendment is proposed to increase the number of young people potentially eligible to take part in the study due to lower than expected rates of young people scoring ≥2 on the CRAFFT screening tool. The entire process of screening, recruitment, completing baseline questionnaire, randomisation and follow up data collection would remain exactly the same as presented in the original application. |
| SA2 | 3.0 | 21/06/2017 | Dr Raghu Lingam | **Amendment 1: Alteration of inclusion criteria**  The amendment concerns the inclusion criteria for the study. Currently, the young person must score >2 on Part B of the CRAFFT form screening tool to be deemed at risk from substance misuse and therefore eligible to participate. We propose that any young person who states they have used substances (including alcohol) within the last 12 months (Part A of the form) is eligible to participate, regardless of their CRAFFT score. The evidence-base on looked after children and care leavers demonstrates that they are a vulnerable group who tend to experience poor outcomes, such as being a higher risk of problematic substance use than their peers. In this sense, being looked after is in itself a risk factor and any substance use further increases their vulnerability and the likelihood of risky behaviour. Also, based on feedback from young people, social workers, foster carers and residential keyworkers, participants are underreporting risky behaviour when being screened by their social workers and thereby excluding themselves from the study as it stands. This is due to a wariness of disclosing such information in the presence of their social worker, despite being able to complete the form confidentially and seal it in an envelope. Young people and carers also report that some social workers are acting as gatekeepers and encouraging the young people to complete the CRAFFT form incorrectly and/or completing it on their behalf.  Broadening the inclusion criteria will increase recruitment rates by allowing more young people to be invited to take part in the study. The rest of the screening and recruitment process will remain the same. |
| SA3 | 4.0 | 26.04.2018 | Professor Eileen Kaner | **Amendment 1: Change of CI:**  The Chief Investigator of this project is now Professor Eileen Kaner, who was originally a co-applicant on the study. NIHR have approved this change. Prof Kaner is in the same Institute and University as the original CI Dr Raghu Lingam. This change is due to Dr Lingam relocating overseas to the University of New South Wales, Australia. Dr Lingam remains a co-applicant on the study.  **Amendment 2: Change of participants for LAC survey:** We propose to make changes to secondary research objective b. regarding the electronically administered survey: b. To conduct an electronically administered survey with LAC service leads across England to characterise usual care locally and nationally, and identify potential collaborative centres for a future multi-centre RCT. An online survey was due to be completed with an identified LAC lead in each English Local Authority area. The survey was planned so as to help us understand usual care provision to LAC who may be using drugs and/or alcohol. A contact number and email was obtained for each LAC lead and a link to the online survey was circulated. The email was followed by a phone call, although the calls often resulted in an alternative name being provided or messages being taken. The research team followed up each contact on three occasions without success. Overall this attempt resulted in only four completed surveys.  We also attended the National Leaving Care Benchmarking Forum at their conference in March 2017, which brought together senior LAC representatives from 80 Local Authorities. A print version of the survey was put in all delegate packs and a pre-paid envelope was included for individuals to return the completed survey. Again, the response rate was poor and we only received two completed surveys.  Feedback identified that: • The contact person didn’t identify themselves as a named LAC lead and suggested that other professionals would be better placed to answer the questions • There were too many questions to answer. We therefore propose to reduce the number of questions on the survey and to contact a manager or senior practitioner within each young person’s drug and alcohol agency within each Local Authority area to discuss the drug and alcohol service offer for LAC. There are 150 services in total to contact.  Therefore, the revised objective would be: b. To conduct an electronically administered survey with Young people’s drug and alcohol service leads across England to characterise usual care locally and nationally, and identify potential collaborative centres for a future multicentre RCT.  Method: Telephone numbers and generic email address for each agency are available online. The research team will contact each agency by email (addressed to a manager and/or senior practitioner) that will include an anonymous link to the survey on Qualtrics. Non-responses will be chased up by telephone and researchers will re-send the email/link if necessary. The simplified questions are attached as a Word document  . **Amendment 3: Alterations to Process Evaluation interviews with LAC:** With regard to qualitative interviews in the Process Evaluation, we now aim to carry out as many interviews with young people as possible (with 20 as a minimum), rather than a purposeful sample of only 20 trial participants. This is due to low recruitments into the trial and the risk of low follow up rates due to the transient nature of this population. This will ensure we have as much data as possible with regard to their experiences of the study. This is encouraged by NIHR. The recruitment process and topic guides remain the same.  **Amendment 4: Staff changes:** Professor Janet Shucksmith and Dr Alison Steele are no longer in post and therefore no longer on the research team. |
| NS1 | 5.0 | 20.03.19 | Professor Eileen Kaner | **Amendment 1: Additional consent form**  A research PPI group was established at the request of LAC attending one of the North East Children in Care Councils. Within this PPI group, a short 5 minute video was produced to be used at dissemination events. When LAC participated in this particular piece of PPI work, the research team adhered to the same process of obtaining informed consent from LAC and their corporate parent as explained in section 4.2.1. However in addition, participants in the research PPI group, also signed an additional consent form due to them producing a video within which their personal image was used. |
