# Supplementary material for: A pilot feasibility randomised controlled trial of two behaviour change interventions compared to usual care to reduce substance misuse in looked after children and care leavers aged 12-20 years: The SOLID study
Source: PLoS One. 2020 Sep 8;15(9):e0238286. doi: 10.1371/journal.pone.0238286 (PMC7478815; doi:10.1371/journal.pone.0238286)
Supplement: S2 File — (DOCX) [file pone.0238286.s002.docx]

**S2: Supporting information file- UKATT process rating scale**

The UKATT process rating scale covers both MET and SBNT and when rating the 26 different items were considered the items were as follows;

1. Maintaining structure
2. Agenda setting
3. Explanation of philosophy of treatment/treatment session
4. Review inter-session change
5. Consistency of problem focus
6. End of session summary
7. Homework
8. Drinking- feedback/negative consequences
9. Alternative activities to drinking
10. Eliciting client concern about drinking
11. Social support for change
12. Eliciting self-efficacy for change
13. Involvement of others in behaviour change
14. Commitment to drinking goal
15. Identifying sources of support for change
16. Ambivalence
17. Creating conflict
18. Eliciting commitment to change drinking
19. Eliciting optimism for change
20. Therapist as task orientated
21. Therapist as active agent for change
22. Reflective listening
23. Collaboration
24. Interpersonal focus
25. Exploration of feeling
26. Empathy.

Each item was rated on the extent to which the practitioners carried out each specific item and the quality of the therapist’s behaviour, using the following scales;

To what extent did the therapist perform the behaviour within each item?

Not at all A little Somewhat Considerably Extensively

0 1 2 3 4

How well did the therapist perform the behaviour within each item?

Not at all well A little Somewhat Considerably Very well

0 1 2 3 4

The research team assessed the extent to which the intervention delivery was true to the therapeutic principles on which it was based.
