# Supplementary material for: A pilot feasibility randomised controlled trial of two behaviour change interventions compared to usual care to reduce substance misuse in looked after children and care leavers aged 12-20 years: The SOLID study
Source: PLoS One. 2020 Sep 8;15(9):e0238286. doi: 10.1371/journal.pone.0238286 (PMC7478815; doi:10.1371/journal.pone.0238286)
Supplement: S3 File — (DOCX) [file pone.0238286.s003.docx]

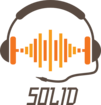


| **Full Title:** | **SOLID (Supporting Looked After Children and Care Leavers In Decreasing Drugs, and alcohol):**  a pilot feasibility study of interventions to decrease risky substance use (drugs and alcohol) and improve mental health of Looked After Children and Care Leavers aged 12 -20 years |
| --- | --- |
| **Short Title/Acronym:** | **SOLID** |
| **Protocol Version Number & Date:** | **Version 1.0**  **23.03.2016** |

Statement:

This protocol has regard for the HRA guidance.

**RESEARCH REFERENCE NUMBERS**

| **IRAS Number:** | 188074 |
| --- | --- |
| **NHS REC Reference:** | **16/NE/0123** |
| **Research Registry & References:** | **ISRCTN TBC** |

**RESEARCH SPONSOR**

| **Sponsor Name:** | **Newcastle University** |
| --- | --- |
| **Sponsor Reference:** | **BH142311** |

**RESEARCH FUNDER(S)**

| **Funder Name:** | **NIHR Public Health Research Programme** |
| --- | --- |
| **Funder Reference:** | **14/183/08** |

**SIGNATURE PAGE**

The undersigned confirm that the following protocol has been agreed and accepted. The Chief Investigator agrees to conduct the trial in compliance with the approved protocol and will adhere to the Research Governance Framework, Good Clinical Practice (GCP) guidelines, the relevant Standard Operating Procedures and other regulatory requirements as applicable.

I agree to ensure that the confidential information contained in this document will not be used for any other purpose other than the evaluation or conduct of the investigation without the prior written consent of the Sponsor.

**Representative of the Research Sponsor**

| **Name:**  **(print)** | **Ms Kay Howes** | | |
| --- | --- | --- | --- |
| **Position:** | Research Manager | | |
| **Signature:** |  | **Date:** | **22.03.2016** |

**Chief Investigator**

| **Name:**  **(print)** | **Dr Raghu Lingam** | | |
| --- | --- | --- | --- |
| **Position** | **Senior Lecturer Community Child Health** | | |
| **Signature:** |  | **Date:** | **23.03.2016** |

**Statistician**

| **Name:**  **(print)** | **Ms Denise Howel** | | |
| --- | --- | --- | --- |
| **Position:** | **Senior Lecturer in Epidemiological Statistics** | | |
| **Signature:** |  | **Date:**21/03/016 |  |

**Trial Coordinator**

| **Name:**  **(print)** | **Hayley Alderson** | | |
| --- | --- | --- | --- |
| **Position:** | **Research Associate** | | |
| **Signature:** |  | **Date:** | **23/03/2016** |

**KEY TRIAL CONTACTS**

| **Chief Investigator** | **Dr Raghu Lingam**  Clinical Senior Lecturer Community Child Health  Institute of Health & Society  Newcastle University  The Baddiley-Clark Building Richardson Road  Newcastle upon Tyne  NE2 4AX   United Kingdom  Telephone: +44 (0)191 208 7045 Fax: + 44 (0) 191 208 6043 |
| --- | --- |
| **Study Coordinator** | **Dr Hayley Alderson**  **Research Associate**  Institute of Health & Society  Newcastle University  The Baddiley-Clark Building Richardson Road  Newcastle upon Tyne  NE2 4AX   United Kingdom  Telephone: +44 (0)191 208 7045 Fax: + 44 (0) 191 208 6043 |
| **Sponsor** | **Ms Kay Howes**  Newcastle University  Faculty of Medical Sciences Framlington Place University of Newcastle upon Tyne NE2 4HH  Telephone: +44 (0)191 208 7460 Fax: + 44 (0) 191 208 8901 |
| **Funder(s)** | **National Institute for Health Research**  Evaluation, Trials and Studies Coordinating Centre University of Southampton  Alpha House, Enterprise Road Southampton SO16 7NS |
| **Collaborators/Co-Investigators** | **Professor Eileen Kaner**  Professor of Public Health and Primary Care Research  Institute of Health & Society  Newcastle University  The Baddiley-Clark Building Richardson Road  Newcastle upon Tyne  NE2 4AX   United Kingdom  Telephone: +44 (0)191 208 7045 Fax: + 44 (0) 191 208 6043  **Dr Ruth McGovern**  Senior Research Associate  Institute of Health & Society  Newcastle University  The Baddiley-Clark Building Richardson Road  Newcastle upon Tyne  NE2 4AX   United Kingdom  Telephone: +44 (0)191 208 7045 Fax: + 44 (0) 191 208 6043  **Professor Alex Copello**  School of Psychology University of Birmingham Edgbaston Birmingham B15 2TT Telephone +[44 (0) 121 414 7414](tel:+44%20%20121%20414%207414)  **Professor Janet Shucksmith**  Professor in Public Health  Health and Social Care  Institute  School of Health & Social Care, Teesside University  Telephone +44 (0) 1642 342750  **Ms Denise Howel**  Senior Lecturer in Epidemiological Statistics  Institute of Health & Society  Newcastle University  The Baddiley-Clark Building Richardson Road  Newcastle upon Tyne  NE2 4AX   United Kingdom  Telephone: +44 (0)191 208 7045 Fax: + 44 (0) 191 208 6043  **Dr Alison Steele,**  Consultant Paediatrician  Newcastle upon Tyne Hospitals NHS Foundation Trust  Telephone: 0191 233 6161  **Dr Paul McArdle**  Consultant Child and Adolescent Psychiatrist  Child and Adolescent, Mental Health Services,  Northumberland, Tyne and Wear NHS  Foundation Trust  Tel: 01670 394 256  Fax: 01670 394 803  **Professor Luke Vale**  Health Foundation Chair in Health Economics  Institute of Health & Society  Newcastle University  The Baddiley-Clark Building Richardson Road  Newcastle upon Tyne  NE2 4AX   United Kingdom  Telephone: +44 (0)191 208 7045 Fax: + 44 (0) 191 208 6043  **Dr Frauke Becker**  Research Associate (health economics)  Institute of Health & Society  Newcastle University  The Baddiley-Clark Building Richardson Road  Newcastle upon Tyne  NE2 4AX   United Kingdom  Telephone: +44 (0)191 208 7045 Fax: + 44 (0) 191 208 6043  **Professor Elaine McColl** Director Newcastle Clinical Trials Unit  Newcastle University 1-2 Claremont Terrace Newcastle upon Tyne NE2 4AE  United Kingdom Phone: +44 (0)191 222 7260 Fax: +44 (0)191 222 8901 |
| **Out of Hours Contacts** | **Dr Raghu Lingam**  University of Newcastle |
| **Committees** | Trial Oversight Committee (TOC).  Chair of TOC (pending approval by NIHR board): Professor Monika Lakhanpaul  Professor of Integrated Community Child Health  Population, Policy and Practice  Institute of Child Health 30 Guilford Street London WC1N 1EH  Dr Doug Simpkiss  University of Warwick  Coventry CV4 7AL |
|  |  |

**TRIAL SUMMARY**

| **Trial Title** | **SOLID (Supporting Looked After Children and Care Leavers In Decreasing Drugs, and alcohol):** a pilot feasibility study of interventions to decrease risky substance use (drugs and alcohol) and improve mental health of Looked After Children and Care Leavers aged 12 -20 years |
| --- | --- |
| **Acronym** | SOLID |
| **Summary of Trial Design** | Pilot feasibility randomised controlled trial |
| **Summary of Participant Population** | Looked After Children and Care Leavers (LAC) aged 12-20 years who screen positive for being at risk of early drug and alcohol use (≥ 2 in the CRAFFT). |
| **Planned Sample Size** | Phase 1 Formative Research  20 1:1 interviews with LAC  20 1:1 interviews with Carers  1 focus group with professionals  1 focus group with Drug and alcohol workers  Pre pilot trials  5 1:1 interviews with LAC trialing the interventions  5 1:1 interviews with Drug and Alcohol workers trialing the interventions  LAC leads survey:  Surveys with all named LAC Community Pediatric leads in each local authority in England (n=152).  Intervention finalization workshop  1 focus group with Professionals  1 focus group with LAC and Carers  Phase 2 Pilot RCT  Sample size required - minimum of 35 LAC per arm at follow-up (3 arm trial). Aim to recruit 150 LAC in total to allow for 30% loss to follow-up.  Process Evaluation  20 1:1 interviews with LAC  20 1:1 interviews with Carers  15 1:1 interviews with Drug and alcohol workers  1 focus group with Social Workers  1 focus group with Drug and alcohol workers |
| **Planned Number of Sites** | 5 (Newcastle, Durham and Teesside (Middleborough, Stockton and Redcar) |
| **Intervention Duration** | 6 sessions over a maximum of 12 weeks |
| **Follow Up Duration** | 12 months (+8 week window) |
| **Planned Study Period** | 24 months |

| **Intervention arms** | 2 active intervention arms:   - 6 sessions of Motivational Enhancement Therapy (MET) or - 6 sessions of Social Behavioural Network Therapy (SBNT)   Interventions will take place over a maximum of a 12 week period. |
| --- | --- |
| **Control arm** | Usual social worker delivered care with additional signposting to local drug and alcohol third sector services. |
| **Inclusion criteria** | - LAC aged 12-20 years - Screen positive for at risk of substance misuse i.e. scoring ≥ 2 on the CRAFFT screening questionnaire - Consents to take part in the study |
| **Exclusion criteria** | - Actively receiving treatment from drug and alcohol services - Unable to access drug and alcohol services e.g. due to imminent move out of area. - Unable to give informed consent due to capacity or language barriers |
| **Outcome of pilot feasibility trial** | Stop/Go criteria have been developed for progression to a definitive trial.  Definite Go (‘green light’) defined as :   - ≥60% of eligible participants consenting to pilot feasibility trial; - ≥ 80% LAC attend ≥ 60% of sessions as planned in a given intervention arm. - Retention of ≥ 70% of consented participants for provision of key outcome data at 12 months. - The intervention can be delivered with fidelity i.e. the content, frequency, duration and quality of the intervention can be delivered as set out in the intervention delivery manual. - An indication from qualitative interview and focus group work that the intervention(s) is (are) perceived as acceptable to both young people and Drug and Alcohol workers.   Definite Stop (‘red light’) defined as :   - < 40% of eligible participants consenting to pilot feasibility trial - < 20% LAC attend ≥ 60% of sessions as planned in a given intervention arm. - retention of < 50% of consented participants for provision of key outcome data at 12 months - Value of information analysis shows that future research is not worthwhile (i.e. the expected value of sampling information (EVSI) < £0. - It is clear from the process data from staff and participants that the intervention procedures have low fidelity in terms of content, frequency, duration and quality and that they are unfeasible to deliver. - An indication from qualitative interview and focus group work that the intervention(s) is (are) not acceptable to LAC and Drug and alcohol workers |
| **Primary Outcomes in definitive trial** | - Episodes of heavy episodic drinking (≥5 units in 1 day) in the preceeding 30 day period - Frequency of use of the most problematic classified substance in preceeding 30 days   (data from Timeline Follow-back questionnaire-30) |
| **Secondary Outcomes in definitive trial** | - Self-reported mental health and wellbeing using the Strength and Difficulties Questionnaire (SDQ) and the Warwick-Edinburgh Mental Well-being scale (WEMWBS). - Self-reported health related quality of life measured using the EQ-5D-5L. - Placement stability for the young person - Self-reported occasions of ‘drunkenness’ in the last 30 days (past 30-day intoxication in ESPAD[^7^](#_ENREF_7)) to compare against the objective standard-drink unit measure. - Self-reported sexual behaviour measured using items taken from the computer assisted self-interview (CASI) questionnaire used in the Avon Longitudinal Study of Parents and Children (ALSPAC) supplemented with questions relating to regret in sexual encounters and unprotected sex used in the ESPAD. - Self-reported antisocial and criminal behaviour measured by a questionnaire used in ALSPAC. - Self-reported use of health and social services using a bespoke questionnaire - Estimates of cost to health and social services - Estimates of quality adjusted life years (QALYs) derived from responses to the EQ-5D 5L |
| **Randomisation procedure** | Individual stratified randomisation, via centralised web-based system, based on placement type (residential, not residential), sites, and age band (<14 and ≥14 and over) |
| **Blinding** | Blinding of group allocation will not be possible for the LAC who will complete the self-report follow up assessment.  Where possible the researchers will not know group allocation. |

# Contents

[Contents 12](#_Toc446424964)

[1. BACKGROUND 17](#_Toc446424965)

[1.1 The Importance of Substance Use in Looked After Children and Care Leavers (LAC) 17](#_Toc446424966)

[1.2 Current Evidence for Interventions to Reduce Substance Misuse 18](#_Toc446424967)

[2. RATIONALE 19](#_Toc446424968)

[2.1 Risk assessment 20](#_Toc446424969)

[3. OBJECTIVES AND OUTCOME MEASURES 21](#_Toc446424970)

[3.1 Aims 21](#_Toc446424971)

[3.2 Objectives 21](#_Toc446424972)

[3.3 Outcome Measures 22](#_Toc446424973)

[3.4 Primary Outcome for Future Trial 22](#_Toc446424974)

[3.5 Secondary Outcomes for Future Trial 23](#_Toc446424975)

[4. STUDY DESIGN & STUDY SETTING 25](#_Toc446424976)

[4.1 Study Setting 25](#_Toc446424977)

[PHASE 1 - FORMATIVE STUDY 26](#_Toc446424978)

[4.2 Intervention Adaptation 27](#_Toc446424979)

[4.2.1 Participant identification and Data Collection 27](#_Toc446424980)

[4.2.2 Data Transcription and Analysis 28](#_Toc446424981)

[4.2.3 Iterative Synthesis and Final Intervention Development 28](#_Toc446424982)

[4.3 LAC Leads Survey 29](#_Toc446424983)

[5. TRIAL PROCEDURES 30](#_Toc446424984)

[5.1 ELIGIBILITY CRITERIA 32](#_Toc446424985)

[5.1.1 Inclusion Criteria 32](#_Toc446424986)

[5.1.2 Exclusion Criteria 32](#_Toc446424987)

[5.2 Recruitment 32](#_Toc446424988)

[5.2.1 Patient Identification 32](#_Toc446424989)

[5.2.2 Screening 32](#_Toc446424990)

[5.2.3 Consent 33](#_Toc446424991)

[5.3 Baseline Assessments 33](#_Toc446424992)

[5.4 Randomisation 33](#_Toc446424993)

[5.5 Follow-up Assessments 33](#_Toc446424994)

[5.6 Blinding 34](#_Toc446424995)

[5.7 Unblinding 34](#_Toc446424996)

[5.7.1 Schedule of Events 35](#_Toc446424997)

[5.8 Withdrawal Criteria 37](#_Toc446424998)

[5.9 Storage and Analysis of Samples 37](#_Toc446424999)

[5.10 End of Trial 37](#_Toc446425000)

[6. TRIAL INTERVENTIONS 38](#_Toc446425001)

[6.1 Name and Description of Interventions 38](#_Toc446425002)

[6.2 Intervention delivery, training and supervision 40](#_Toc446425003)

[6.3 Known Risks 41](#_Toc446425004)

[6.4 Concomitant Medications & Therapies 41](#_Toc446425005)

[7. ASSESSMENT OF COMPLIANCE/ PROCESS EVALUATION 42](#_Toc446425006)

[7.1 Data Collection 43](#_Toc446425007)

[7.2 Data Analysis 43](#_Toc446425008)

[8. Study Timeline 46](#_Toc446425009)

[9. SAFETY REPORTING 46](#_Toc446425010)

[9.1 Definitions 46](#_Toc446425011)

[9.2 Recording and Reporting AEs and SAEs 47](#_Toc446425012)

[9.3 Recording and Reporting USARs 49](#_Toc446425013)

[9.4 Responsibilities 49](#_Toc446425014)

[9.5 Notification of Deaths 50](#_Toc446425015)

[9.6 Reporting Urgent Safety Measures 50](#_Toc446425016)

[10. STATISTICAL CONSIDERATIONS 51](#_Toc446425017)

[10.1 Analysis Population 51](#_Toc446425018)

[10.2 Statistical Analyses 51](#_Toc446425019)

[10.2.1 Analysis of the Primary Outcome Measures 51](#_Toc446425020)

[10.2.2 Analysis of Secondary Outcome Measures 51](#_Toc446425021)

[10.3 Sample Size Calculation 51](#_Toc446425022)

[11. ECONOMIC EVALUATION 52](#_Toc446425023)

[11.1 Overview 52](#_Toc446425024)

[11.2 Methods 52](#_Toc446425025)

[11.2.1 Data Collection 52](#_Toc446425026)

[11.2.2 Data Analyses 52](#_Toc446425027)

[12. DATA HANDLING 54](#_Toc446425028)

[12.1 Data Collection Tools and Source Document Identification 54](#_Toc446425029)

[12.2 Data Handling and Record Keeping 54](#_Toc446425030)

[12.3 Access to Data 54](#_Toc446425031)

[12.4 Archiving 55](#_Toc446425032)

[13. MONITORING, AUDIT & INSPECTION 56](#_Toc446425033)

[14. ETHICAL AND REGULATORY CONSIDERATIONS 57](#_Toc446425034)

[14.1 Research Ethics Committee Review and Reports 57](#_Toc446425035)

[14.2 Peer Review 57](#_Toc446425036)

[14.3 Public and Patient Involvement 57](#_Toc446425037)

[14.4 Regulatory Compliance 57](#_Toc446425038)

[14.5 Protocol Compliance 58](#_Toc446425039)

[14.6 Notification of Serious Breaches to GCP and/or the Protocol 58](#_Toc446425040)

[14.7 Data Protection and Patient Confidentiality 58](#_Toc446425041)

[14.8 Indemnity 58](#_Toc446425042)

[14.9 Amendments 59](#_Toc446425043)

[14.10 Post-Trial Care 59](#_Toc446425044)

[14.11 Access to the Final Trial Dataset 59](#_Toc446425045)

[15. DISSEMINATION POLICY 60](#_Toc446425046)

[16. REFERENCES 61](#_Toc446425047)

[17. APPENDICES 65](#_Toc446425048)

[17.1 Appendix 1 - Safety Reporting Diagram 65](#_Toc446425049)

[17.2 Appendix 2 – Amendment History 66](#_Toc446425050)

**GLOSSARY OF ABREVIATIONS**

| **ABBREVIATION** | **DEFINITION** |
| --- | --- |
| AE | Adverse Event |
| AR | Adverse Reaction |
| AUDIT | Alcohol Use Disorders Identification Test |
| CI | Chief Investigator |
| CRF | Case Report Form |
| CRAFFT | Car, Relax, Alone, Forget, Friends, Trouble(s). |
| DALY | Disability adjusted life year |
| EVSI | Expected value of sampling information |
| ENG | Expected net gain |
| GCP | Good Clinical Practice |
| ICF | Informed Consent Form |
| ISF | Investigator Site File |
| ISRCTN | International Standard Randomised Controlled Trials Number |
| LAC | Looked After Children and Care Leavers |
| MET | Motivational Enhancement Therapy |
| NCTU | Newcastle Clinical Trials Unit |
| NHS | National Health Service |
| NPS | Novel Psychoactive substances |
| OR | Odds ratio |
| PI | Principal Investigator |
| PIC | Participant Identification Centre |
| PIS | Participant Information Sheet |
| QA | Quality Assurance |
| QALY | Quality Adjusted Life Years |
| QC | Quality Control |
| R&D | Research & Development |
| RCT | Randomised Control Trial |
| REC | Research Ethics Committee |
| SAE | Serious Adverse Event |
| SAR | Serious Adverse Reaction |
| SBNT | Social Behavioural Network Therapy |
| SDQ | Strengths and Difficulties Questionnaire |
| SDV | Source Data Verification |
| SOP | Standard Operating Procedure |
| SSI | Site Specific Information |
| USAR | Unexpected Serious Adverse Reaction |
| TLFB-30 | Timeline Follow Back data covering the last 30 days |
| TMG | Trial Management Group |
| TOC | Trial Oversight Committee |
| TMF | Trial Master File |

**TABLES AND FIGURES**

[Table 1 RCT showing Schedule of Events 35](#_Toc446411932)

[Table 2 Contrasts between MET and SBNT 38](#_Toc446411933)

[Table 3: Specification of the Process Evaluation 45](#_Toc446411934)

[Figure 1: Component parts of the SOLID study phase 1 26](#_Toc446411853)

[Figure 2 Component parts of the SOLID study phase 2 with trial flow diagram 31](#_Toc446411854)

[Figure 3 BDI Theory of change model for MET 39](#_Toc446411855)

[Figure 4 BDI Theory of change model for SBNT 40](#_Toc446411856)

[Figure 5 Study Timeline SOLID Project Gantt chart 46](#_Toc446411857)

# BACKGROUND

## The Importance of Substance Use in Looked After Children and Care Leavers (LAC)

Drug and alcohol (substance) use in young people is a major public health problem, which causes a significant economic strain on the NHS and society.[^1^](#_ENREF_1) Substance use accounts for 11% of the total burden of disease, calculated as disability adjusted life years lost, in high income countries.[^2^](#_ENREF_2) It was estimated in 2013 that alcohol related harm costs the UK £21 billion annually with an additional £15.4 billion estimated to result from drug addiction.[^3^](#_ENREF_3) Risky substance use in adolescence predicts adult alcohol and drug use and significantly increases the risk of adult mental health disorders, crime and poverty.[^4-6^](#_ENREF_4) Although there has been an overall fall in drug use in teenagers over the last decade, the UK is still in the top five for lifetime use of cannabis and other illicit drugs in 15 to 16 year olds and the top ten with regards to binge drinking (heavy sessional or risky single occasion drinking) in the last 30 days across 36 European countries.[^7^](#_ENREF_7) In addition to cannabis use, there has been an explosion in the use of novel psychoactive substances (NPS) in the UK, commonly known as ‘legal highs’, which are likely to have significant physical and mental health consequences.[^8^](#_ENREF_8)^,^ [^9^](#_ENREF_9)

In 2014, one in every 200 children and young people (CYP) in England, a total of 69,000, were looked after by local authority services, a rise of 7% in 5 years.[^10^](#_ENREF_10) Looked After Children and Care Leavers (LAC) are those children and young people up to the age of 21 who have been placed under the legal care of local authorities. The 2014 NICE guidelines, ‘Interventions to reduce substance misuse among vulnerable young people’, highlighted LAC as a vulnerable group to substance use.[^11^](#_ENREF_11) About 7% of the approximately 21,000 young people accessing specialist drug and alcohol services self-reported that they were in care.[^12^](#_ENREF_12) Unfortunately, there is limited research, including cost effectiveness data, and, at present no national guidelines, on the most effective interventions to decrease risky drug and alcohol use in this group. This lack of data was highlighted by the CMO’s annual report 2012, which stated that one of the key research areas was to assess the most effective interventions to reduce multiple risk taking behaviour, including drug and alcohol use in this group.[^13^](#_ENREF_13) LAC have multiple risk factors for substance use, poor mental health, school failure and early parenthood.[^14^](#_ENREF_14) These factors include parental poverty, absence of support networks, parental substance misuse, poor maternal mental health, early family disruption and, in the majority of cases, abuse and/or neglect.[^15^](#_ENREF_15)^,^ [^16^](#_ENREF_16) LAC, aged 11 to 19 years, have a 4 fold increased risk of drug and alcohol use than children not in care.[^17^](#_ENREF_17) Twenty-five percent of LAC aged 11 to 19 years and 42% of young people in residential care drank alcohol at least once a month, compared to 9% of young people not looked after.[^17^](#_ENREF_17) A national survey of care leavers showed that 32% smoked marijuana daily and data from 2012 showed 11.3% of LAC aged 16-19 years had a diagnosed substance use problem.[^18^](#_ENREF_18)^,^ [^19^](#_ENREF_19) In addition, LAC have a nearly fivefold increased odds of at least one mental health diagnosis including anxiety, depression or behavioural disorders (OR: 4.92; 95% CI: 4.13, 5.85) than their non-LAC peers, further increasing their risk of substance misuse and poor life chances.[^20^](#_ENREF_20)

The long term outcome of LAC in terms of health, education, employment and risk of criminality is poor, resulting in a significant cost to society and increased risk of intergenerational poverty. Forty percent of 20 year olds who have been in the care system are not in education, employment or training (NEET).[^19^](#_ENREF_19) Data from the 1960 British birth cohort at age 30 years showed that men with a history of being in public care were twice as likely to be unemployed (OR: 2.6; 95% CI: 1.4, 5.0), have a criminal conviction (OR: 2.3; 95% CI: 1.5, 3.4) and have been seen for a mental health, drug or alcohol related problem after the age of 16 years (OR: 1.7; 95%CI: 1.1, 2.6) than non-looked after peers.[^21^](#_ENREF_21) There are limited longitudinal data looking at the impact of drug and alcohol use on young people as they transition to adulthood. However, research from the criminal justice system in Scotland showed that 34% of youth offenders had been in care. Of these offenders, 75% reported drug usage (compared to 57% of those not previously in care).[^22^](#_ENREF_22) Effective interventions in LAC could have a beneficial effect on the long term mental and physical health of these vulnerable young people, importantly reduce health inequality and, due to their increased risk of early parenthood, potentially impact intergenerational health.

## Current Evidence for Interventions to Reduce Substance Misuse

There is evidence that preventative interventions (school based, family based and mixed input) can reduce alcohol use in young people.[^23-26^](#_ENREF_23) Foxcroft’s review of motivational interviewing (MI) for young adults found insufficient evidence to recommend this approach to reduce alcohol use. However, many of the included studies were primary prevention intervention studies, brief (15 minutes) or single session interventions.[^27^](#_ENREF_27) In contrast, a recent systematic review and meta-analysis found that early intervention with MI was effective in reducing substance use in early substance using young people, especially in studies that delivered multiple sessions of individual MI.[^26^](#_ENREF_26)

There is increasing focus on family and social network therapies as a way of engaging and supporting hard to reach children. Programmes such as the ‘Strengthening Families Programme for Parents and Youth 10-14’, currently being evaluated in the UK, emphasise family connectedness and may enhance resilience and prevent multiple risk taking behaviours.[^28^](#_ENREF_28) Family based therapy, including multidimensional family therapy and brief strategic family therapy, have been shown to be effective in reducing alcohol usage in young people.[^29^](#_ENREF_29) However, many family-based approaches are complex to deliver and therefore offer limited scope for implementation at scale. As LAC are living outside their biological family unit, this creates a challenge for family-based approaches but not necessarily wider social network approaches.

There is little UK evidence on the best way to identify children in the early ‘at risk stage’ of substance use or the most effective intervention regimes for delivery within current resources, especially when considering the most vulnerable teenagers such as LAC.[^30^](#_ENREF_30) A recent US RCT of a multicomponent intervention in girls in foster care targeting both the young person’s self-efficacy and the carers’ parenting skills showed significantly lower levels of substance use (smoking, marijuana and alcohol usage) in the intervention group compared to the control, though baseline difference between groups were not controlled for. There is an urgent need for robust evidence to inform treatment guidelines to decrease substance use in this high risk population of young people.

# RATIONALE

Although the risk of substance misuse in LAC is well documented, service provision for this group is poor, with only 41% of those young people with substance use problems receiving help.[^31^](#_ENREF_31) Given the vulnerability to early onset substance use within LAC populations, secondary prevention interventions, which target young people who are already using substances may be more effective, cost effective, and salient to the individuals receiving it than universal, primary prevention approaches.

NICE guidelines recommend multiple sessions of motivational interviewing or family based support or group based behavioural therapy over 1 to 2 years to reduce substance use in high risk young people.[^11^](#_ENREF_11) Due to the complexity and length of these interventions they are not feasible to be delivered at scale. The current study will adapt and evaluate the pilot feasibility of two alternative evidence based counselling interventions: Motivational Enhancement Therapy (MET) – a concentrated form of MI developed within the NIH MATCH study;[^32^](#_ENREF_32) and Social Behavioural Network Therapy (SBNT) – an approach drawing from family and social interventions in substance use.[^33^](#_ENREF_33)^,^ [^34^](#_ENREF_34) Both interventions involve counselling but focus on different behaviour change pathways (illustrated in Figures 1 and 2), with internal thoughts and views shaping the decisional-balance in MET compared with external, social influences in SBNT.

For many LAC it is the experience of abuse or neglect from within their families that has led them to Local Authority care and may account for the high rates of psychiatric morbidity in this population.[^20^](#_ENREF_20) The absence of a supportive family unit has been associated with increased rates of substance misuse in young people.[^35^](#_ENREF_35) SBNT has been designed to mobilise and develop support networks which are wider than just biological family and include peers; it has been found to be effective in reducing substance misuse in adults when delivered through routine services.[^36^](#_ENREF_36) Interventions based on SBNT have the potential to address this central vulnerability within a LAC population. Details of the intervention packages are given in Section 6.

In phase 1 of the research project, the intervention packages based on these two counselling approaches will be adapted collaboratively with LAC, carers and professionals. We hope to develop interventions that LAC will engage with and that can be integrated into their care pathway and will consider novel ways to tackle the emerging health issue of NPS use. The pilot evaluation will give insights into the acceptability of the interventions and feasibility of a definitive multicentre RCT. Specifically, the process evaluation will provide rich data on both interventions including “dose” delivered (how many sessions taken up), its fidelity (assessment of content and interaction), acceptability (LAC and practitioner perceptions about receiving and delivering the actual interventions) and the potential “mechanism of change” (whether our theory of change pathways are borne out in practice) from both the perspective of the service provider and LAC (service user). These data will allow us to assess if either of the interventions is clearly more acceptable to both service users and providers, is most likely to be integrated into routine service delivery, and to elicit behaviour change in the young person. These data along with feedback from LAC and practitioners will be used to draw element of the interventions together into a single ‘optimised’ intervention if appropriate. MET targets internal thoughts and motivations, whilst SBNT uses the external social network of the young person as a catalyst for change. In the substance use field there is a tradition of different interventions all yielding small positive effects in head to head trials. Thus we will explore if the approaches are best considered separately or if there are positive ground to combine them in to a single ‘optimized’ intervention approach to take forward as an enhanced treatment intervention, designed for LAC, to the definitive trial. Effective and efficient interventions in this group could have a beneficial effect on long term mental and physical health of these vulnerable young people, reduce health inequality and due to their increased risk of early parenthood, impact intergenerational health.

## Risk assessment

| **Trial Categories based upon the potential risk associated with the intervention** |  |
| --- | --- |
| ***Type A****:* Comparable to that of the standard medical care | Trials involving non-pharmacological therapies if knowledge derived from controlled trials already exists.{UKATT Research Team, 2005} |

# OBJECTIVES AND OUTCOME MEASURES

## Aims

The SOLID pilot feasibility trial (Supporting Looked After Children and Care Leavers In Decreasing Drugs, and alcohol) aims to assess the pilot feasibility and acceptability of a definitive three-arm multi-centre randomised controlled trial (two behaviour change interventions and care as usual) to reduce risky substance use (illicit drugs and alcohol), and improve mental health in Looked After Children and Care Leavers (LAC aged 12 -20 years). The study will take place in multiple sites in the North East of England and will have two linked phases: 1. Formative study phase, followed by 2. Pilot feasibility randomised controlled trial (RCT).

## Objectives

Primary Objectives

Phase 1 Formative Study: To adapt two behaviour change interventions for Looked After Children (LAC) and care leavers to help reduce risky substance use: i, Motivational Enhancement Therapy (MET); ii. Social Behavioural Network Therapy (SBNT). This will be carried out with LAC and care leavers, their carers, drug and alcohol workers, and LAC social workers to ensure acceptability and feasibility of the intervention packages.

Phase 2 Pilot Feasibility RCT: To conduct a three arm pilot RCT (comparing: i. MET, ii. SBNT, and iii. Control – usual care), to determine if rates of eligibility, recruitment and retention of LAC, and acceptability of the interventions are sufficient to recommend a definitive multi-centre randomised controlled trial.

Secondary research objectives:

Phase 1 Formative Study:

1. To refine the intervention packages for integration into LAC care pathways.
2. To conduct an electronically administered survey with LAC service leads across England to characterise usual care locally and nationally, and identify potential collaborative centres for a future multi-centre RCT.

Phase 2 Pilot Feasibility RCT:

1. To establish response rates, variability of scores, data quality and acceptability of the proposed outcomes measures for self-reported alcohol and drug use, health related quality of life, mental health and well-being, sexual behaviour and placement stability 12 months post recruitment, in order to inform a sample size calculation for a definitive multicentre RCT.
2. To assess acceptability, engagement and participation with the MET and SBNT based interventions by LAC, their carers and front line drug and alcohol workers.
3. To carry out a process evaluation to include fidelity of intervention delivery and qualitative assessment of the barriers to successful implementation; and to assess if key components from the MET and SBNT interventions can be combined to develop a new optimised intervention.
4. To develop cost assessment tools, assess intervention delivery costs and carry out a value of information analysis to inform a definitive study.
5. To apply pre-specified ‘stop: go’ criteria and determine if a definitive multi-centre randomised controlled trial is feasible, and, if so, develop a full trial protocol.
6. To consider findings from the study as a whole in order to develop a core intervention delivery package, potentially of a single optimised intervention, linked to a theory of change model to use in the definitive trial.

## Outcome Measures

As a pilot feasibility trial, the primary outcomes of the pilot RCT will be rates of recruitment and retention of LAC at 12 month follow up; along with acceptability and engagement with both the interventions and trial procedures. However, the pilot feasibility trial will also examine the response rates, acceptability and pilot feasibility of outcomes, **measured at 12 months post enrolment** that would be used in a definitive trial. The proposed primary and secondary outcomes for a definitive trial are outlined in Sections 3.4 and 3.5 respectively.

## Primary Outcome for Future Trial

Our study ultimately aims to decrease both alcohol and drug use in LAC; as such the proposed primary outcomes of the definitive trial, to be rehearsed in the pilot feasibility trial, will be heavy episodic drinking and frequency of use of the most problematic classified substance. Heavy episodic drinking (high intensity ‘binge’ drinking) in the preceding 30 day period will be derived from Timeline Follow Back data covering the last 30 days (TLFB-30) [^37^](#_ENREF_37) and defined as the 'number of occasions where 5 or more standard drink units are consumed on a single drinking day' as used in the ESPAD survey.[^7^](#_ENREF_7) This measure has been chosen as an objective measure of likely intoxication or ‘drunkenness’ which in turn is associated with behavioural risk taking. The most problematic classified substance (excluding alcohol) will be defined using the multi-criteria decision analysis hierarchy developed by Nutt et al.[^38^](#_ENREF_38); we will again use data from TLFB-30. While the most prevalent drugs for the general population of under 18 year olds are alcohol and cannabis, NPS use is becoming increasingly common.[^9^](#_ENREF_9) NPS will be classified along with mephedrone. The TLFB-30 will be compared to first 7 days of TLFB and the shorter AUDIT, ASSIST and CRAFFT tools[^39^](#_ENREF_39) in terms of data yield, pilot feasibility and acceptability as part of planning for the future multi-centre RCT. This group of young people that have often had to fill out paper work in terms of their LAC status and findings from our PPI work have highlighted the importance of respondent fatigue. Longer instruments may lead to respondent burden and incomplete data. The study needs to assess the accuracy but also the efficiency of the tools we use. As such data yield from the TLFB -7, and the shorter AUDIT, ASSIST and CRAFFT will be compared to the TLFB-30.

## Secondary Outcomes for Future Trial

Secondary outcomes to be assessed in the future trial, and rehearsed in the pilot feasibility trial, will be:

- Self-reported mental health and wellbeing measured using the Strength and Difficulties Questionnaire (SDQ) and the Warwick-Edinburgh Mental Well-being scale (WEMWBS). [^40^](#_ENREF_40)^,^ [^41^](#_ENREF_41)
- Self-reported health related quality of life measured using the EQ-5D-5L.[^42^](#_ENREF_42)
- Self-reported occasions of ‘drunkenness’ in the last 30 days (past 30-day intoxication in ESPAD[^7^](#_ENREF_7)) to compare against the objective standard-drink unit measure.
- Placement stability for the young person – as part of the 12 month follow up questionnaire, questions will be developed and administered around placement stability. In addition consent will be obtained to link to social services records to assess longer term placement stability.
- Self-reported sexual behaviours measured using items taken from the computer assisted self-interview (CASI) questionnaire used in the Avon Longitudinal Study of Parents and Children (ALSPAC) supplemented with questions relating to regret in sexual encounters and unprotected sex used in the ESPAD.[^7^](#_ENREF_7)^,^ [^43^](#_ENREF_43)
- Self-reported antisocial and criminal behaviour measured by a questionnaire used in ALSPAC. [^44^](#_ENREF_44)
- Self-reported use of health and social services using a bespoke questionnaire
- Estimates of cost to health and social services
- Estimates of quality adjusted life years (QALYs) derived from responses to the EQ-5D 5L

The Strengths and Difficulties Questionnaire (SDQ) is a standardised screening questionnaire used extensively in mental health research with young people and used previously with LAC.[^17^](#_ENREF_17) It consists of 25 questions arranged to create four difficulty subscales (measuring emotional symptoms, conduct, hyperactivity and inattention and peer relationship difficulties) and a measure of pro-social behaviour. The 4 difficulty subscales are summed to give a “total difficulty score” out of 40.[^40^](#_ENREF_40)

The Warwick-Edinburgh Mental Well-Being Scale (WEMWBS) is a 14 item scale of mental well-being covering subjective well-being and psychological functioning, in which all items are worded positively and address aspects of positive mental health. The tool has been used extensively with adults and has recently been validated for use in teenagers.[^41^](#_ENREF_41)

The EQ-5D-5L is a self-completion questionnaire, comprising 5 dimensions of quality of life, each with 5 response options. Responses to the EQ-5D-5L will be used to calculate quality adjusted life years (QALYs).[^42^](#_ENREF_42)

The computer assisted self-interview (CASI) was used at several time-points from age 12 years to assess romantic and sexual activities in young people in ALSPAC, a UK longitudinal birth cohort study.[^43^](#_ENREF_43) Questions relating to enjoyment of sexual experience will be replaced by two questions from the ESPAD multi-country survey of alcohol and drug use assessing regret in engagement in sexual contacts and engagement in unprotected sexual intercourse, particularly relevant in relation to sexual encounters preceded by substance misuse.[^7^](#_ENREF_7) The advantage of the CASI is that it is graded, with more intimate sexual contact not asked about if lesser contact such as kissing and cuddling has not yet been experienced. This instrument has been discussed with the study PPI group who acknowledged the graded nature of administration and supported its use. As in ALSPAC a computerised version will be administered.

A questionnaire on antisocial behaviour will be used, this has previously used in ALSPAC and is derived from questions used in The Edinburgh Study of Youth Transitions and Crime.[^45^](#_ENREF_45)

# STUDY DESIGN & STUDY SETTING

The proposed research has two linked phases: 1. Formative phase consisting of adaptation and manualisation of the interventions, and a national survey of LAC Clinical Service Leads to help characterise usual care across England and identify potential collaborative centres for a definitive trial. This will be followed by a second phase consisting of a pilot feasibility randomised controlled trial (RCT). This second phase of the project will also have a detailed economic component outlined in section 11.

The two phases are detailed in Sections 5 and 6 respectively and are illustrated in Figures 1 and 2 on pages 26 and 31 respectively

## Study Setting

The research will be set in three sites in the North East of England; local authorities in Newcastle, Teesside, and County Durham, which are covered by the Fuse public health research network. The North East of England is an area of increased health and social care need and has the highest rates of poverty in the country with 24% of households living below the poverty line. The region is however not uniform and encompasses a mixture of urban, periurban and semi-rural areas. The percentage of black and ethnic minority groups across the region also varies from 10% in Newcastle to 2% in Durham. Teesside, Newcastle and Durham have 112, 102 and 60 LAC per 100,000 children respectively, the 7th, 15th and 83rd highest rates of any area in the country (out of 152). Each area has a mixture of child placement type’s i.e. residential homes, extended family and foster care placement. The project will also include a survey of LAC health leads across England utilising the Clinical Research Network (Children) and links with the Royal College of Paediatrics to identify collaborative centres to develop a multicentre definitive RCT.

# PHASE 1 - FORMATIVE STUDY

Figure 1: Component parts of the SOLID study phase 1

North East Fuse Network: Newcastle, Durham and Teesside.

National

Formative Research with young people Formative Research with professionals Pre-pilot feasibility trials Survey

**Drug and Alcohol worker** identify LAC and carers eligible to be contacted by the research team.

[TIPs YP initial contact and assent form provided- Doc 9]

Researcher identifies LAC Social workers and Drug and Alcohol Workers

**Social workers** identify LAC and carers eligible to be contacted by the research team.

[YP initial contact and assent form provided- Doc 1]

National survey of LAC services

Intervention finalisation workshop

Workshop 1: LAC Young people and carers and Workshop 2: Key stakeholders, drug and alcohol workers, LAC Representatives, Local authority leads, National representatives.

Pre-pilot trials of interventions followed by:

1:1 interviews with LAC young people receiving the interventions [Participant assent/consent and parent/guardian consent- Doc 13 or 14]

1:1 interviews with Drug and Alcohol workers trialling the intervention [Participant consent form- Doc 15]

**Consent/assent not given**- LAC not enrolled into study continues with usual care

Professional not enrolled into study

**Consent/Assent given**

1. Researcher contacts LAC & parent/guardian and carer informed- consent/assent requested

2. Researcher contacts Drug and Alcohol workers- Participant information leaflet provided [Doc 12] and informed consent requested.

**Assent not given-** LAC not enrolled into study continues with usual care

**Assent given**- Participant information leaflet sent out to YP and parent/guardian [Doc 10 or 11]

Focus Group Discussion with Social Workers and Drug and alcohol workers

[Participant consent form- Doc 7]

**Consent not given-** Professionals not enrolled into study

Consent/Assent given

Researcher contacts LAC Social Workers and Drug and Alcohol Workers- Informed consent requested

**Consent not given**- Professionals not enrolled into study

**Verbal consent given**- Participant information leaflet [Doc 8] sent out to professionals

1:1 interviews with LAC young people

[Participant assent/consent and parent/guardian consent- Doc 5 or 6]

1:1 interviews with Carers

[Participant consent form- Doc 7]

**Consent/assent not given**- LAC not enrolled into study continues with usual care

**Consent/Assent given**

Researcher contacts LAC & parent/guardian and carer informed- consent/assent requested

**Assent not given**- LAC not enrolled into study continues with usual care

**Assent given**- Participant information leaflet sent out to YP [Doc 2 or 3] and parent/guardian [Doc 4]

## Intervention Adaptation

We will use a systematic[^46^](#_ENREF_46) and person-based approach[^47^](#_ENREF_47) to intervention adaptation and refinement. We shall retain essential therapeutic elements of the interventions (SBNT and MET) but refine aspects of content and delivery for our target group of Looked After Children and Care Leavers as well as exploring acceptability in an iterative and age appropriate manner. Eliciting the views of our target group (LAC) and tailoring input to their specific needs is an important part of intervention development; making the intervention more salient and delivery more feasible. Interview guides will be developed with the SOLID PPI group to identify key determinants and behaviours that to be targeted by the proposed interventions.

### **Participant identification and Data Collection**

Young people (LAC and Carer Leavers) will be identified by social workers from their caseload in the research sites (Newcastle, Durham and Teesside).The sample will be chosen to ensure diversity with regards to age, ethnicity, exposure to Drug and alcohol services and placement type. The social worker will share a brief initial contact leaflet with the young person. The social worker will then request written assent for the young person to be approached formally by the study team. The young person under 16 will be seen with an accompanying adult (parent, carer, social worker, children’s home lead) and they will be asked to provide informed assent. For those young adults 16 years and over, informed consent will be taken directly. If the accompanying adult does not have parental responsibility (PR) the research team will contact the adult with PR to obtain informed consent. If the parent is not contactable or it is a risk for the young person for their parent to be contacted in the view of the designated social worker, the social worker/local authority guardian with PR will be contacted to sign the consent form. Information on the study will be shared with parents/carers as appropriate. Written informed assent/consent will be obtained for all participants by research workers.

After informed consent has been given, 1:1 qualitative interviews will be undertaken with a purposive sample of LAC aged 12 -20 years recruited from the LAC service. Interviews will be carried out by trained researchers in the young person’s home or convenient private location to ensure safety of both the young person and researcher. The young person will be given a choice of whether they want to be accompanied by a trusted adult in these interviews who will be there as an observer. Interviews will continue until saturation; however, we envisage approximately 20 1:1 interviews with LAC.

Participants will be remunerated for their time (£10 cinema or equivalent voucher). All interviews will be digitally audio-recorded and transcribed verbatim. Interview guides, informed by psychological theory of change models and developed with the SOLID PPI group, will explore beliefs around drug and alcohol usage and the requirements of any new service. Key behavioural issues and motivational domains as well as the challenges that the intervention should address will be identified.

Separate interviews will be carried out with the carer (foster carer/ family member/ residential worker) that the young person (LAC) interviewed is staying with (n=20). Additional carers will be identified by the social worker to ensure diversity of sample in terms of age, ethnicity and carer type (i.e. foster carer/ family member/ residential worker). Again informed consent from the adult and assent of the young person under 16 years will be taken.

Focus groups will be held with LAC social workers and specialist young people’s drug and alcohol workers. The LAC social workers have key knowledge of the context of LAC as well as many of the ethical issues which must inform intervention development. Informed consent will be obtained from all participants.

In summary we envisage 20 1:1 interviews with LAC, 20 1:1 interviews with their carers, and two focus groups, 1 with social workers and 1 with drug & alcohol workers consisting of approximately 6-8 people per group.

### **Data Transcription and Analysis**

Data transcription will be carried out verbatim by the researchers who undertook the interviews, by project admin support or University approved transcription services. Transcripts will be anonymised by removing names of any individuals mentioned in the course of the interview; a participant key will be stored separately. Data analysis will focus on understanding internal and external drivers of behaviour and also on views about intervention focused on promoting well-being and self-care in early life. Analysis will be an iterative process, using the constant comparative method[^48^](#_ENREF_48), derived from grounded theory, and informed by Bourdieu’s (1990)[^49^](#_ENREF_49) concept of habitus; i.e. we will explore the unconscious beliefs and socialised norms that shape behaviour in LAC, an approach used successfully by this team in qualitative work with young people within the age range of this study.[^50^](#_ENREF_50) Components of the logic models (behaviours, determinants and intervention components), illustrated in Figures 3 and 4, on pages 39 and 40 respectively, will be explored with respondents to further refine the theory of change pathway and clarify intervention delivery issues. Qualitative software (NVIVO 10) will aid in the organisation of thematic codes and categories. These data and our analysis will be used to refine SBNT and MET approaches so they are responsive to the needs and views of substance using LAC. This may include enhancing SBNT core discussion topics with optional topics or highlighting important motivational domains which may be explored within the sessions.

### Iterative Synthesis and Final Intervention Development

Intervention components will be tested with a small group of LAC (n=5) within the Drug and alcohol service to further refine the intervention (pre pilot feasibility trials). Assent will be obtained by the Drug and alcohol worker for the LAC to be approached by the research team. Informed consent and assent as appropriate (i.e. depending on the age of the LAC, as described above) will be obtained from the LAC and their parent/ guardian. Qualitative interviews will then be used in an iterative process with these LAC (n=5) to examine whether the new versions of the interventions are engaging and acceptable to LAC. Interviews will also be carried out with the Drug and alcohol workers (n=5) trialling these intervention components. Key findings will be shared with participants in two intervention development workshops to finalise the intervention adaptation. As such, the interventions will be adapted collaboratively with LAC themselves as well as their carers, and service providers.

## LAC Leads Survey

Each local authority in England has a statutory named LAC clinical lead. Lists of these lead clinicians will be obtained from the British Association of Community Child Health and the Royal College of Paediatrics and Child Health (RCPCH), accessed by Lingam and Steele who are members of these bodies. Using the NIHR Clinical Research Network (Children) we will contact local delivery managers to cross check LAC lead clinicians in each centre with these lists. The named LAC Community Paediatric leads in each local authority in England (n=152) will be contacted by email and letter to complete an online survey in Qualitrics to map drug and alcohol usual care using a questionnaire based on the Template for Intervention Description and Replication (TIDieR) checklist.[^51^](#_ENREF_51) Non-respondents will be identified against our definitive list and contacted by phone to complete the survey with a researcher. This survey will accurately map services and pathways of usual care for LAC and identify potential collaborative centres for a definitive multisite RCT.

# TRIAL PROCEDURES

A three arm pilot feasibility randomised controlled trial will be conducted in three sites in the North East of England: Newcastle, Durham and Teesside, to assess the acceptability of the adapted interventions (SBNT and MET) and the feasibility of taking one or both of the interventions to a full scale multicentre randomised controlled trial. Details of the flow of participants through the study is presented in Figure 2.

Figure 2 Component parts of the SOLID study phase 2

North East Fuse Network: Newcastle, Gateshead, Durham, Middlesbrough, Stockton and Redcar.

**Social worker** screening of all LAC 12-20 years for significant drug/ alcohol use and meeting inclusion criteria (LAC ages 12-20 years, admitted substance use in past 12 months and consents to take part in the study).

Request for assent to contact research team.

[Young people initial contact, assent and CRAFFT- Doc 16]

**Consent/assent not given**- LAC not enrolled into study continues with usual care

**Assent given and inclusion criteria met-** Participant information sheet sent out to YP and parent/guardian.

[Participant information leaflet YP 12-14 or YP 15-20- Doc 17 or 18: sent out by SOLID Admin lead]

Usual care + signposting - services

Drug and alcohol service delivered

Motivational Enhancement Therapy

Drug and alcohol service delivered

Social Behavioural Network Therapy

S

**Researcher Visit 2. Follow-up Evaluation** 12 months post enrolment:

*Substance use (TLFB substance use/30 day, AUDIT and ASSIST)

*Mental health and wellbeing (SDQ, WEMWBS) *Quality of life (EQ-5D) * Sexual behaviour (CASI & ESPAD questions) *antisocial and criminal behaviour *placement stability and *use of health and social services.

**Economic tool development and value of information analysis**

**Ongoing: Process evaluation** -mixed methods including: trial recruitment and retention, intervention adherence and satisfaction: **STOP/GO criteria assessed**

**SOLID Admin Lead**: completes stratified randomisation & group allocation. Admin lead contacts;

1. LAC, and carer with the outcome of allocation;

2. Drug and alcohol services for those allocated to intervention arms.

**Researcher visit 1**

**Consent/Assent given**

**Baseline data collection**. LAC to complete electronic data collection form.

Baseline data collection including *demographics, *placement type, *drug and alcohol use (AUDIT/ASSIST), *mental health and well-being (baseline SDQ and WEMWBS) * Self-reported drunkenness and *health-related quality of life (EQ-5D-5L).

**Researcher visit 1**

1. Inclusion/exclusion criteria checked
2. Informed consent/assent requested [Participant assent/consent and parent guardian consent- Doc 19 or 20]

## ELIGIBILITY CRITERIA

### Inclusion Criteria

Young people will be included in the pilot feasibility trial if they meet all the following inclusion criteria:

- Looked Children and Care Leavers aged ≥12 and ≤20 years
- Screen positive for being at risk of substance misuse i.e. scoring ≥2 on the CRAFFT
- Informed consent given: LAC under 16 years consent from parent/guardian (local authority) and assent from young person; LAC 16 years and over consent from young person.

### Exclusion Criteria

Young people will be excluded from the trial if they are:

- Already in active treatment with drug and alcohol services
- Unable to access drug and alcohol services e.g. due to imminent move out of area.
- Unable to give informed consent in English.

It should be noted that the intervention is delivered in English. The numbers of young people excluded due to language barriers will be reviewed for the definitive RCT.

## Recruitment

### Patient Identification

All Looked After Children and Care Leavers (LAC) aged 12-20 years in the study sites will be identified by their social workers for screening from Local authority lists.

### Screening

LAC within the service are already questioned regarding their drug and alcohol use as part of routine care, however, the tools used are not standardised. Services in the area have agreed to screen all LAC aged 12-20 years for drug and alcohol use by a social worker using the validated 6 question CRAFFT and for anonymised data to be processed in Newcastle. This will ensure that data is accurately recorded and summary statistics can be fed back to each local authority. The CRAFFT has been used extensively with young people, and is sensitive and specific to identify problem substance use.[^39^](#_ENREF_39) Information leaflets introducing the study will be shared with the young person by the social worker. As part of clinical care, anonymised information will be processed centrally; this will include age, gender and locality. Written assent will be obtained from all LAC aged 12-20 for the young person and their parent/ guardian to be contacted by the research team depending on the CRAFFT score. Those scoring ≥2 in the CRAFFT (‘at risk of substance misuse’) will be contacted by the research team if written assent for contact has previously been given to the social worker. Young people that are in active treatment with drug and alcohol services will not be screened as they will not meet eligibility criteria. A screening log will be kept by the social worker to ensure all LAC aged 12 to 20 years who are not in active treatment with drug and alcohol services have the opportunity to be enrolled into the study.

### Consent

Information leaflets for the trial will be sent by the Trial Admin Lead to all potential participants who agree to be contacted, prior to the individual meeting the researcher. The researcher will then contact the young person and their parent/guardian by phone to arrange a convenient time and location to meet. The young person under 16 will be seen with an accompanying adult (parent, carer, social worker, children’s home lead) and asked to provide informed assent. For those young adults 16 years and over, informed consent will be taken directly. If the accompanying adult does not have parental responsibility (PR) the research team will contact the adult with PR to obtain informed consent. If the parent is not contactable or it is a risk for the young person for their parent to be contacted in the view of the designated social worker, the social worker/ local authority guardian with PR will be contacted to sign the consent form. Information on the study will be shared with parents/carers.

## Baseline Assessments

After informed consent has been obtained, the researcher will collect baseline information from the LAC using an electronic questionnaire. Questionnaires will be in an electronic format programmed into mobile tablets which will be handed to the young person. The researcher will be available to answer questions of clarification if needed. This questionnaire will record demographics, placement type, drug and alcohol usage (AUDIT/Alcohol, Smoking and Substance Involvement Screening Tool (ASSIST) [^52^](#_ENREF_52)), mental health and well-being (baseline SDQ and WEMWBS) and health-related quality of life (EQ-5D-5L). Details of the tools used are presented in section 7.1.

## Randomisation

Individual randomisation to the three trial arms will be stratified by placement type (residential/ non-residential), site and age band (12-14/ over 14), to reflect risk profile for substance usage. The randomisation procedure will be carried out by the Trial Admin Lead using the Newcastle Clinical Trials Unit online randomisation service. The Trial Admin Lead will then distribute five letters:

1. Letter to the young person outlining group allocation.
2. Letter to parent outlining study enrolment.
3. Letter to carer outlining study enrolment.
4. Letter to GP outlining study enrolment.
5. Letter to the Drug and alcohol service outlining group allocation and requesting an initial appointment.

Group allocation will be documented by the Trial Admin Lead ensuring that researchers and statisticians are blinded to group allocation. The LAC will be contacted by the drug and alcohol service for treatment to the appropriate treatment group (MET or SBNT) within 6 weeks.

## Follow-up Assessments

All young people enrolled into the trial study will be contacted by phone and letter/email 12 months post recruitment to complete a follow up electronic questionnaire. A recruitment window of 8 weeks will be in place to maximise follow up.

The questionnaire will be administered by the study research associate who will visit the young person in their home/ convenient location. If after repeated attempts the researcher is not able to contact the young person for a face to face follow up, a telephone follow up will be offered. As in the baseline questionnaire, data will be collected using electronic data capture forms programmed onto digital tablets. The researcher will hand the LAC the tablet and be present to answer questions as necessary. The Time line Follow back will be researcher administered.

The data to be collected are listed in Section 3.4 and 3.5. In summary these data are:

- Time Line Follow Back 30 day:
  - Episodes of heavy episodic drinking (≥5 units in 1 day) in the preceeding 30 day period
  - Frequency of use of the most problematic classified substance in preceeding 30 days
- AUDIT and ASSIST
- Mental health and wellbeing: Strength and Difficulties Questionnaire (SDQ) and the Warwick-Edinburgh Mental Well-being scale (WEMWBS).
- Quality of life measured using the EQ-5D-5L.
- Placement stability for the young person
- Sexual behaviour: taken from ALSPAC the computer assisted self-interview (CASI) with questions relating to regret in sexual encounters and unprotected sex used in the ESPAD.
- Antisocial behaviour questionnaire previously used in ALSPAC.
- Use of health and social services using a bespoke questionnaire

In addition, process evaluation data will be collected as presented in Section 7.

## Blinding

Blinding of group allocation will not be possible for the LAC who will complete the self-report follow up assessment or for those delivering the intervention. The study statistician and health economist will be blind to group allocation and will only have details of participant by study number. However, as this is a pilot feasibility trial, the SOLID researchers will be carrying out both process and follow up evaluation so may become aware of study allocation as the trial progresses.

## Unblinding

The trial statistician and health economist will only be unblinded after the final analysis or if requested to do so due to safety concerns expressed by the Trial Oversight Committee.

### Schedule of Events

Table 1 RCT showing Schedule of Events

| **Procedures** | **Visits (insert visit numbers as appropriate)** | | | | | | | | |
| --- | --- | --- | --- | --- | --- | --- | --- | --- | --- |
|  | **Screening** | **Baseline (pre randomisation)** | **Intervention Phase 6 sessions over maximum of 12 weeks** | | | | | | **12 month follow up from baseline** |
|  |  |  | Session 1 | Session 2 | Session 3 | Session 4 | Session 5 | Session 6 |  |
| Informed consent |  | X |  |  |  |  |  |  |  |
| Demographics |  | X |  |  |  |  |  |  |  |
| Placement type |  | X |  |  |  |  |  |  |  |
| Eligibility assessment | X | X |  |  |  |  |  |  |  |
| CRAFFT | X |  |  |  |  |  |  |  |  |
| Randomisation |  | X |  |  |  |  |  |  |  |
| Intervention (MET or SBNT) |  |  | X | X | X | X | X | X |  |
| Self-reported Drunkenness |  |  |  |  |  |  |  |  | x |
| AUDIT |  | X |  |  |  |  |  |  | X |
| ASSIST |  | X |  |  |  |  |  |  | X |
| SDQ |  | X |  |  |  |  |  |  | X |
| WEMWBS |  | X |  |  |  |  |  |  | X |
| EQ-5D-5L |  | X |  |  |  |  |  |  | X |
| TLFB-30 |  |  |  |  |  |  |  |  | X |
| Placement Stability questions |  |  |  |  |  |  |  |  | X |
| ALSPAC CASI + ESPAD questions on sexual behaviour |  |  |  |  |  |  |  |  | X |
| Antisocial / criminal behaviour questionnaire |  |  |  |  |  |  |  |  | X |
| Use of health and social services |  |  |  |  |  |  |  |  | X |
| Adverse event assessments |  | X | X | X | X | X | X | X | X |

## Withdrawal Criteria

Participants have the right to withdraw from the trial at any time without having to give a reason. The team will try to ascertain the reason for withdrawal and document this reason within the Case Report Form.

The Investigator may discontinue a participant from the trial at any time if the Investigator considers it necessary for any reason including:

- Participant withdrawal of consent
- Investigator’s discretion that it is in the best interest of the participant to withdraw
- An adverse event that requires discontinuation of the trial intervention or renders the participant unable to continue in the trial
- Termination of the trial by the sponsor

Participants who withdraw from the trial will not be replaced.

Participants may withdraw from study intervention and/or from follow-up data collection. Participants requesting withdrawal from intervention will be asked if they would be willing to remain in the study for purposes of follow-up data collection as per schedule of events. Data provided to the point of withdrawal will be retained.

## Storage and Analysis of Samples

No biological samples will be collected as part of this study. Identifiable data will be destroyed after 10 years.

## End of Trial

The end of the trial will be considered as the last participant’s follow-up visit at 12 months.

# TRIAL INTERVENTIONS

## Name and Description of Interventions

The adapted interventions based on MET and SBNT will be delivered by existing drug and alcohol team workers.[^32^](#_ENREF_32)^,^ [^33^](#_ENREF_33) These workers will be specifically trained to deliver the study interventions. The key areas of contrast between MET and SBNT are summarised in Table 2.

Table 2 Contrasts between MET and SBNT

(taken from Copello et al 2009 with permission from the author)

| MET | SBNT |
| --- | --- |
| The individual’s motivation is central to addiction process. An increase in the individual’s motivation to change will increase likelihood of success | Positive social support for change is central to addictive process |
| Aim to alter the individuals decisional balance and to amplify discrepancy between current use of alcohol and future goals | Aims to maximise outside positive support for change |
| The therapist discusses motivation as seen by the client | Therapist is an active agent for change within the problem drinkers social environment |
| Significant others play some role in treatment but are not central | The Therapist always attempts to engage significant others that are central to the behaviour change process |
| The therapist elicits reasons for change from the “problem drinker” using a number of techniques | The therapist acts as a team leader, leading the team to the attainment of specific goals. |

Behaviour Determinants Intervention (BDI)[^53^](#_ENREF_53) theory of change models for MET and SBNT are presented in Figures 3 and 4 respectively on pages 39 and 40. Both models explain the proposed change pathways for the interventions, highlighting the determinants for change and key behaviours targeted.

**Motivational Enhancement Therapy (MET)** is a client centred, directive counselling approach developed within the NIH MATCH study as a concentrated version of motivational interviewing which adds a problem feedback component to standard treatment. [^32^](#_ENREF_32)^,^ [^54^](#_ENREF_54) The intervention has been shown to decrease substance use in a range of participants including adolescents.[^34^](#_ENREF_34)^,^ [^54^](#_ENREF_54) The basic assumption of MET is that the motivation and responsibility for change lie within the client and it is the therapist’s role to create an environment to enable the client to change. Unlike cognitive behavioural interventions, ambivalence is assumed to be the norm and motivation is formed and enhanced within the context of the therapist-client relationship. The therapist employs specific strategies to develop motivation, seeking to mobilize the client’s inner resources and intrinsic motivation and in doing so, enables the client to initiate and achieve behaviour change.

The therapist supports the client to explore their substance use and elicits their own concerns and motivations. By reflecting and reaffirming this ‘change talk’,[^55^](#_ENREF_55) a discrepancy is developed between where the client currently is and where they want to be; tipping the decisional balance in favour of change. Motivation is further built through self-motivational statements, strengthening the commitment to change, which is negotiated through a plan for change. Personal responsibility for change is encouraged. This is in contrast to cognitive behavioural approaches which teach clients the skills needed to change. Within the follow-through phase, the therapist’s role is to review progress that the client has made against their self-chosen goals and strategies. Motivation to change is renewed by this process and self-efficacy is reaffirmed.[^32^](#_ENREF_32)

Figure 3 BDI Theory of change model for MET

**
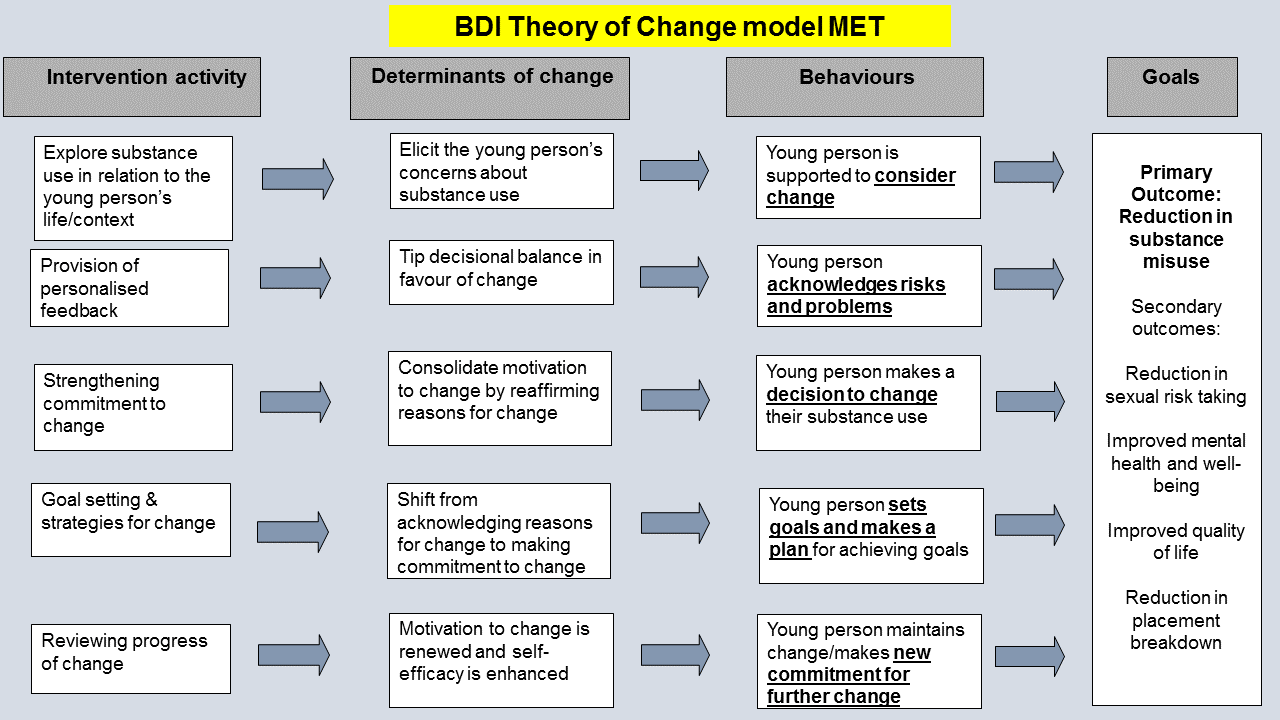
**

**Social Behavioural Network therapy (SBNT)** is a systematic counselling approach, which utilises cognitive and behavioural strategies to help clients build social networks supportive of positive behaviour change in relation to problem substance misuse and goal attainment.[^56^](#_ENREF_56) SBNT is based on the premise that social network support for change is key in helping people deal effectively with addictive behaviour. The intervention focuses on addressing substance use by engaging with a network of positive support for lifestyle change. This work is conducted in collaboration with the young person with whom early identification of the social network is carried out. An important aspect of SBNT, especially important for LAC, is that it aims to sustain engagement with vulnerable young people by widening the reach of the intervention beyond the traditional family to include supportive peers or other important figures as perceived by the young person including e.g. teachers, social workers or possibly wider family members such as grandparents.

The theoretical basis of the approach is informed by evidence of the positive influence of social support and support for behaviour change in improving substance use. Alcohol treatment research in adult populations has shown that general social support, alcohol-specific social support and the drinking behaviour of the social network members have all been shown to be unique predictors of positive alcohol treatment outcomes.[^56-59^](#_ENREF_56)

Informed by previous research, the intervention aims to integrate strategies found to be effective in other family and network approaches and, is based on the concepts of identifying, engaging with and enhancing social support for a positive change in substance use. SBNT consists of a series of core and elective topics used within sessions and illustrated in the theory of change model (Figure 3). Core strategies include identifying young people’s social networks, improving communication and coping mechanisms, and given the nature of substance misuse, developing a network-based relapse management plan. The therapeutic approach also has scope to address client-focussed elective areas, for example educational requirements or, when relevant, other important areas such as risk taking.

SBNT has been shown to be effective and cost effective in reducing alcohol usage in harmful adult drinkers.[^36^](#_ENREF_36)^,^ [^60^](#_ENREF_60) On-going work with one of the participating services in the proposed application (Drug and Alcohol Service- Drug and alcohol Newcastle)[^61^](#_ENREF_61) has shown that SBNT is well received by young people aged 12 to 18 who are not in the LAC system.

Figure 4 BDI Theory of change model for SBNT

**
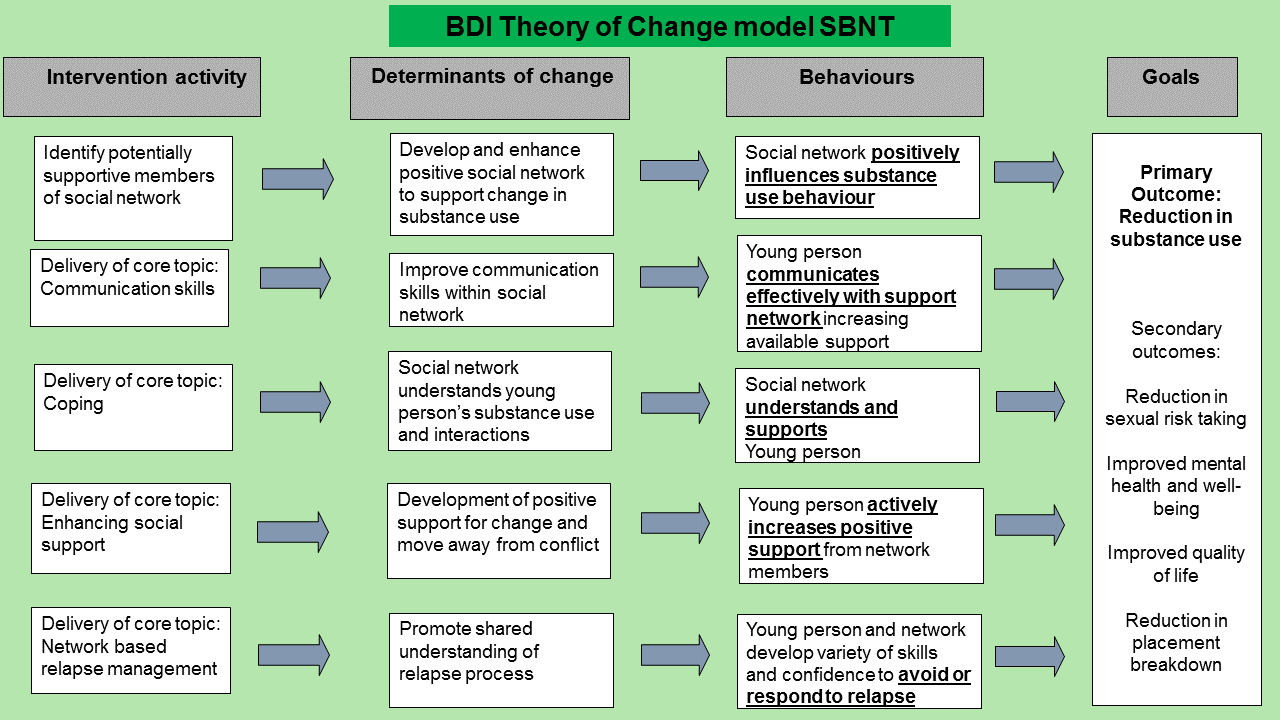
**

## Intervention delivery, training and supervision

Each research site will have a control and two separate intervention teams trained to deliver either the MET or SBNT intervention. For example, in Newcastle (the smallest drug and alcohol team) as part of the preparatory work for the application, it has been agreed that the existing team of six people will be split into three teams of two workers each. Two workers will be trained by the research team to use MET, two will be trained to use SBNT and two workers (control) will have no additional training. Provision will be made for leave. The MET and the SBNT sub teams will have separate external intervention-specific supervision delivered by the research team. A similar method to minimise contamination has been used by the team as part of the HTA funded Y-SBNT study.[^36^](#_ENREF_36) Possible residual contamination of co-workers residing in the same premises, will be explored within the process evaluation before full trial.

We aim to offer 6 sessions of SBNT or MET, with each session lasting 50 mins. In both intervention arms, sessions will be offered weekly to fortnightly, with a maximum period of 12 weeks. The rationale for this number of sessions stems from learning from the UKATT trial and pilot work using SBNT with young people referred to child and adolescent mental health services. The final number of sessions offered will be finalised as part of the formative research. We will assess the number of sessions offered and actually attended by LAC to determine the appropriate number of sessions in a definitive trial. We will incorporate to feedback on duration, content, and location of sessions in the final specification of our intervention to ensure the intervention is acceptable to LAC and staff and can be delivered at scale by existing services.

## Known Risks

Based on previous experience we do not anticipate any significant harm or consequences to result from the interventions. All relevant risk assessments and responses to risk protocols from the participating services will be adhered to. In terms of social harms, the particular focus of the social intervention is on positive support, using a range of strategies to identify those people in the young person’s social environment that could offer this type of positive support. If the interventions uncover unmet medical or psychological need, onward referral to appropriate services will be initiated.

## Concomitant Medications & Therapies

Young people already in active treatment with drug and alcohol services will be excluded from the study as outlined in Section 5.1.2 and will be noted in the trial flow diagrams.

# ASSESSMENT OF COMPLIANCE/ PROCESS EVALUATION

The process evaluation aims to understand and document the key lessons learned from implementing SOLID (both the interventions and the trial processes) and to evaluate factors needed to deliver the intervention at scale. It will use a mixed methods approach (collecting both quantitative and qualitative data), based on the framework outlined by Steckler and Linnan (2002)[^62^](#_ENREF_62) but enhanced to include an emphasis on the mechanism of change at the level of the service provider (implementation, embedding and integration of the interventions – informed by Normalisation Process Theory) and behaviour change at the level of the individual young person (LAC).

The evaluation will also specifically explore contamination through 1:1 interviews with LAC and the Drug and alcohol worker. Core components of the evaluation are presented in Table 3 on page 45.

We will use Carroll’s (2007) definition of fidelity i.e. we will assess whether intervention sessions are delivered as planned in terms of content, frequency, duration and coverage [^63^](#_ENREF_63). These largely quantitative measures, derived from attendance records, will be supplemented by detailed assessment of audio-taped sessions and evidence from qualitative interviews and focus groups with Drug and Alcohol workers and managers. We propose to assess the quality of intervention delivery (treatment fidelity) by applying a validated process rating scale (UKATT PRS)[^64^](#_ENREF_64), developed in the UKATT trial. All sessions will be audio recorded and a 20% random sample of SBNT and MET sessions will be analysed ensuring we sample early, mid and late sessions of both interventions. UKATT PRS specifically covers MET and SBNT and assesses items including commitment, optimism, collaboration and interpersonal focus which help determine if the LAC are actively engaged in the intervention sessions. To make this assessment, the two research associates will independently rate the sampled audio-taped intervention sessions covering the 1 to 6 sessions for MET and SBNT.

Interview schedules for staff will be constructed to highlight the 4 core concepts of Normalisation Process Theory [^65^](#_ENREF_65):

1. *Coherence:* a shared understanding of the work **(do the workers understand the aims and the logic behind the intervention?)**
2. *Cognitive participation*: a shared agreement and engagement with the techniques of the work **(do the workers ‘buy into’ and ‘own’ the aims and logic behind the intervention and the ways in which it is being implemented?)**
3. *Collective action:* agreement with the organisation of the work **(what do the workers and managers do in practice to make the intervention work in their setting for themselves and the client group?)**
4. *Reflexive monitoring:* assessment and monitoring of the work **(are the workers engaged enough to be able to suggest improvements to the logic or practice of the intervention?)**.

This theory driven process evaluation will allow us to understand the extent to which staff understand the principles and core components of the interventions, value and believe in them, make adaptations to their usual work plan to ensure the interventions are delivered successfully and reflect on ways which they can improve delivery of the service. The analysis will inform our understanding of the mechanism of change from a practitioner perspective and consider key barriers to successful delivery and integration of the interventions at the level of the system.

Along with mechanism of change from the perspective of the service provider, we will collect qualitative data from LAC and their carers to understand how the interventions have affected the LAC themselves and how they would recommend changing the interventions to make them more effective. We will specifically probe around the proposed determinants of change illustrated in the BDI theory of change models (Figures 1 and 2, on pages 26 and 31 respectively). These data will allow us to refine these models.

## Data Collection

As highlighted in Table 3, on page 45, we will collect both quantitative and qualitative data to assess each of the process evaluation questions. Quantitative data will include a review of attendance records. Consent for access to participant records will be taken during initial consenting for the trial along with permission to potentially be contacted as part of the process evaluation. Qualitative data will be collected from a purposive sample of LAC (aiming for a maximum diversity sample – sampling criteria will be gender, placement type, study arm and age), their carers, social workers and Drug and alcohol workers. Data collection will continue until saturation; however as a minimum we will carry out 20 qualitative interviews with LAC, 20 interviews with carers, fifteen interviews with Drug and alcohol workers and two focus groups one with social workers and one with members from the drug and alcohol teams. Qualitative contacts will be preceded by informed consent. Interviews will take place within 3 months of intervention delivery. As outlined, all MET and SBNT sessions will be recorded and a 20% random sample will be independently rated by two researchers using the UKATT PRS. The UKATT PRS will be trialled and adapted as part of the pre-pilot feasibility trials in the formative research phase. Consent for this part of the study again will be taken at trial enrolment, however, the Drug and alcohol worker will make it clear that the young person can refuse to audiotaping of their session if they wish – this will not affect their participation in the study as a whole.

## Data Analysis

Qualitative data will be analysed thematically based on the constant comparison approach.

Where more than one source of data is collected for a given research question, findings will be triangulated e.g. for fidelity, we will explore content of sessions and probe on key aspects of input (qualitative interview data from LAC; log book entry plus focus group data from Drug and alcohol workers), frequency and duration (questionnaire data to be completed by Drug and alcohol services and verified by the researcher against service records) and coverage (assessment of the number of sessions offered and received, along with equity of intervention delivery by age, placement type and geographic coverage). Triangulation enhances the validity of conclusions by exploring the convergence, complementarity and dissonance of results on related research questions obtained from different methodological approaches, sources, theoretical perspectives, or researchers.[^66^](#_ENREF_66)

In this case we will use methodological triangulation, data triangulation and investigator triangulation. The range of both quantitative and qualitative data will be brought together using a ‘triangulation protocol’. [^67^](#_ENREF_67)^,^ [^68^](#_ENREF_68)This involves identifying themes or threads from each data source and method, and then sorting these into similar categories. These are then ‘convergence coded’ to identify where there is agreement, silence and dissonance in terms of data from different sources and methods.

Table 3: Specification of the Process Evaluation

| Process evaluation component | Key research questions | Additional information | Data source |
| --- | --- | --- | --- |
| **Recruitment and reach** | i. Has SOLID been able to recruit LAC (numbers screened, numbers screened positive, number consenting to participate in the study)? | Equity in terms of by age, placement type and geography. | Drug and alcohol services records, trial screening logs, consent forms, records of who was retained, and records of when and if possible why participants dropped out of the study. |
|  | ii. Was recruitment equitable across demographic groups |  |  |
| **Dose delivered** | iii. Were intervention sessions offered to young people?  iv. What proportion of these sessions were completed? |  |  |
| **Fidelity** | v. Were the interventions delivered as planned? | Fidelity defined by Carroll (2007) i.e. content, frequency, duration and coverage. | Content: Qualitative interview data from LAC;  Log book entry plus focus group data from Drug and alcohol workers. |
|  |  |  | Frequency and duration: questionnaire data to be completed by Drug and alcohol services and verified by the researcher against service records |
|  |  |  | Coverage: Drug and alcohol service records number of sessions offered and received. |
|  | vi. To what extent have the new interventions been integrated into routine practices? | Guides developed based on normalisation theory to probe: coherence, cognitive participation, collective action and reflexive monitoring (May and Finch 2009). | Qualitative focus group discussions plus interviews with Drug and alcohol workers. |
| **Quality (treatment fidelity)** | vii. Were the interventions delivered with quality? | UKATT Process rating Scale: 7 items for MET and 8 items for SBNT to measure quality (treatment fidelity) | Researcher analysis of audio recording of a random 20% subsample of intervention sessions using UKATT process rating scale for MET and SBNT. |
| **Dose received/ acceptability** | vii. Was the intervention acceptable to LAC? If not why not? |  | Qualitative interviews with LAC |
| **Retention in the trial** | viii. What was the proportion of young people recruited into the trial who were retained till 12 month follow up and what measures can increase trial retention? |  | Trial records of recruitment and retention.  Qualitative interview data from LAC |
| **Contamination** | ix. To what extent did Drug and alcohol workers know about and were influenced by SBNT/ MET also being undertaken in their hub? |  | Qualitative interviews and focus group discussions with Drug and alcohol workers and managers. |
|  | ix. To what extent did LAC know about and were influenced by SBNT/ MET interventions being offered to other LAC that they knew? |  | Qualitative interviews and focus group discussions with Drug and alcohol workers and managers. |
| **Mechanism of change** | x. What were the facilitators and barriers to integration of the interventions into the Drug and alcohol service? | Normalisation theory components coherence, cognitive participation, collective action and reflexive monitoring (May and Finch 2009). | Qualitative interviews and focus group discussions with Drug and alcohol workers and managers. |
|  | xi. Did the interventions make a difference to the lives of LAC and did they alter their behaviour? Are the BDI models appropriate for the mechanism of change? |  | Qualitative interviews LAC and their carers. |

# Study Timeline

Figure 5 Study Timeline SOLID Project Gantt chart

|  | Months | 1- 3 | 4 - 6 | 7- 9 | 10 -12 | 13- 15 | 16- 18 | 19 -21 | 22 -24 |
| --- | --- | --- | --- | --- | --- | --- | --- | --- | --- |
| Phase 1 Formative study | Qualitative data collection and analysis |  |  |  |  |  |  |  |  |
|  | Pre-pilot feasibility trials |  |  |  |  |  |  |  |  |
|  | Intervention finalisation workshops |  |  |  |  |  |  |  |  |
|  | On Line Survey of LAC leads |  |  |  |  |  |  |  |  |
|  | Formative research report |  |  |  |  |  |  |  |  |
| Phase 2 Pilot RCT | Recruitment |  |  |  |  |  |  |  |  |
|  | Intervention delivery |  |  |  |  |  |  |  |  |
|  | Process evaluation |  |  |  |  |  |  |  |  |
|  | Economic tool development and VOI analysis |  |  |  |  |  |  |  |  |
|  | Follow up data collection |  |  |  |  |  |  |  |  |
|  | Analysis/writing up/dissemination |  |  |  |  |  |  |  |  |
| Study steering Committee (SSC) meetings | |  |  |  |  |  |  |  |  |
| Dissemination events | |  |  |  |  |  |  |  |  |

# SAFETY REPORTING

## Definitions

| Term | Definition |
| --- | --- |
| Adverse Event (AE) | Any untoward medical occurrence in a participant, including occurrences which are not necessarily caused by or related to the intervention under study. |
| Adverse Reaction (AR) | An untoward or unintended response in a participant which is related to the intervention under study i.e. that a causal relationship between the trial intervention and an AE is at least a reasonable possibility and the relationship cannot be ruled out.  All cases judged by either the reporting medically qualified professional or the Sponsor as having a reasonable suspected causal relationship to the trial intervention qualify as adverse reactions. |
| Serious Adverse Event (SAE) | A serious adverse event is any untoward medical occurrence that:   - Results in death - Is life-threatening* - Requires inpatient hospitalisation or prolongation of existing hospitalisation - Results in persistent or significant disability/incapacity - Other important medical events that jeopardise the participant or require intervention to prevent one of the above consequences   * - life-threatening refers to an event in which the participant was at immediate risk of death at the time of the event; it does not refer to an event which hypothetically might have caused death if it were more severe. |
| Serious Adverse Reaction (SAR) | An adverse event that is both serious and, in the opinion of the reporting Investigator, believed with reasonable probability to be due to the trial intervention, based upon the information provided. |
| Unexpected Serious Adverse Reaction (USAR) | A serious adverse reaction, the nature and severity of which is not consistent with the known information about the intervention under study. |

## Recording and Reporting AEs and SAEs

All AEs and SAEs occurring from the time of randomisation in the Pilot RCT until follow up is completed (12 months post enrolment) must be reported. Depending on the nature of the event, the reporting procedures below should be followed. Any questions concerning adverse event reporting should be directed to the Chief Investigator or the Study Coordinator (Research Associate) in the first instance.

**Adverse Event (AEs):** All non-serious adverse events (as defined in section 9.1 above and including non-serious adverse reactions) during study participation must be recorded on an adverse event/harms case report form and sent to the Study Coordinator within 2 weeks of the event. Severity of AEs will be graded on a three-point scale (mild, moderate or severe). The Study Coordinator will enter the data onto the study database. In addition, the Study Coordinator will contact the LAC team manager monthly to ensure no adverse events are missed.

Expected AEs include the following:

- Severe drug and alcohol use
- Mental health difficulties
- Self-harm and suicidal ideation

**Serious Adverse Event (SAEs):** All SAEs (as defined in section 9.1 above and including serious adverse reactions) during study participation must be reported to the Chief Investigator within 24 hours of the site becoming aware of the event. The initial report should ideally be made by completing the study SAE form and sending via the fax number listed on page 64 of the protocol. Alternatively, if this is not possible, the initial report may be made by telephone to the number listed on page 64 of the protocol. In the case of incomplete information at the time of initial reporting, all appropriate information should be provided as follow-up as soon as this becomes available. Relationship of the SAE to study procedures will be assessed by the Chief Investigator, as will the expected or unexpected nature of the SAE.

As the study deals with a vulnerable population, expected SAEs include those related to the following which result in hospitalisation:

- Severe drug and alcohol use
- Mental health difficulties
- Self-harm and suicidal ideation

SAEs exclude:

- any pre-planned hospitalisations (e.g. elective surgery) not associated with clinical deterioration
- routine treatment or monitoring of the studied indication, not associated with any deterioration in condition
- elective or scheduled treatment for pre-existing conditions that did not worsen during the study

For each SAE the following information will be collected:

- Full details in medical terms and case description
- Event duration (start and end dates, if applicable)
- Action taken
- Outcome
- Seriousness criteria
- Causality in the opinion of the investigator
- Whether the event is considered expected or unexpected (decision to be made by reference to the expected SAEs listed above **only**).

Any change of condition or other follow-up information should be reported to the Study Coordinator (Research Associate) as soon as it is available, or at least within 24 hours of the information becoming available. Events will be followed up until the event has resolved or a final outcome has been reached.

## Recording and Reporting USARs

All USARs (serious adverse reactions classified as unexpected, as defined in section 9.1) occurring after each participant’s first session of the intervention until their follow up is completed (12 months post enrolment) will be reported to the NHS REC. The CI will perform this reporting.

The assessment of expectedness will be performed by the CI against the known information for the trial.

USARs must be reported no later than 15 calendar days after the CI has first knowledge of the event. Any relevant follow-up information should be sought and reported as soon as possible after the initial report.

As soon as a site suspects that a SAR may be a USAR they must contact the CI, sponsor representative and the research associate immediately by completing an SAE form, and sending it via the fax number listed on page 64 of the protocol.

The reporting timeframe starts at day 0 when the CI is in receipt of a minimum set of information:

- Sponsor trial reference and trial name (sponsor reference)
- Patient trial number and date of birth
- Name of intervention
- Date of notification of the event
- Medical description of the event
- Date and time of the onset of the event (including event end date if applicable)
- Causality assessment
- Seriousness of the event, particularly if life threatening or fatal
- An identifiable reporter (e.g. Principal Investigator)

All sites are expected to fully cooperate with the CI in order that a full and detailed report can be submitted to the NHS REC within the required timelines.

PIs will be informed of all USARs by the CI.

## Responsibilities

***Chief Investigator***

- *Clinical oversight of the safety of trial participants, including an ongoing review of the risk/benefit.*
- *Using medical judgement in assigning seriousness, causality and expectedness of SAEs where it has not been possible to obtain local assessment.*
- *Using medical judgement in assigning expectedness to SARs*
- *Immediate review of all USARs.*
- *Review of specific SAEs and SARs in accordance with the trial risk assessment and protocol.*
- *Checking for AEs and ARs when participants attend for treatment or follow-up*
- *Ensuring that all SAEs and SARs, including USARs, are recorded and reported to the Sponsor within 24 hours of becoming aware of the event and provide further follow-up information as soon as available.*
- *Ensuring that AEs and ARs are recorded and reported to the Sponsor in line with the requirements of the protocol.*
- *Expedited reporting of USARs to the REC within required timelines*
- *Notification to all investigator sites of any USAR that occurs*

***Sponsor***

- *Review of any USARs*

***TOC***

- *Review of safety data collected to date to identify any trends*

## Notification of Deaths

All deaths will be reported to Sponsor irrespective of the cause of death.

## Reporting Urgent Safety Measures

An Urgent Safety Measure (USM) is an action that the Sponsor or an Investigator may take in order to protect the subjects of a trial against any immediate hazard to their health or safety. Upon implementation of an USM by an Investigator, the Sponsor and CI must be notified immediately and details of the USM given.

All members of the research team are responsible for reporting (to the Sponsor or delegated party) if they believe that the implementation of safety measures is required. The following procedure should be followed if the Study Coordinator (Research Associate) becomes aware of information that indicates an immediate change in a trial procedure, or that a temporary halt to a trial may be necessary in order to protect trial participants from any immediate hazard to their health and safety:

The Study Coordinator (Research Associate) should immediately record the details of the incident on the current version of the NCTU incident report form. They should then inform the CI, Sponsor (via their designated representative), and/or other designated individual (such as nominated senior NCTU personnel, who has been delegated the responsibility for decision making with regard to urgent safety measures), with full details of the information they have received relating to the incident.

The individual designated to make a decision about implementing safety measures (CI) will consider whether urgent safety measures are necessary to protect participants against any immediate hazard. If trial participants are at risk, the designated individual should inform the NCTU that urgent safety measures are to be implemented so that proposed actions can be agreed.

Verbal notification, via telephone, should then be provided to the main REC and the outcome of these discussions should be summarised on the NCTU incident report form.

The requirement to initiate an urgent safety measure, and the proposed corrective/preventative action, should be communicated to all sites immediately (i.e. within 24 hours of the decision) with acknowledgment of its receipt and implementation received from each PI.

The implemented urgent safety measures (e.g. amendment to protocol, temporary halt to the trial or premature closure of the trial) must be reported by the CI, on behalf of the Sponsor, in writing to the REC within **three days**.

Arrangements for contacting the individual responsible for decision-making with regard to urgent safety measures during extended breaks (for example Christmas and Easter) should be made, to ensure that appropriate cover is in place.

# STATISTICAL CONSIDERATIONS

## Analysis Population

All analyses will be conducted on an intention to treat basis, with sensitivity analyses used to investigate the impact of removing individuals who did not receive the interventions as allocated.

## Statistical Analyses

As a pilot feasibility trial, the main analyses will be descriptive, in order to inform the design of a future definitive study.

### Analysis of the Primary Outcome Measures

The primary outcomes are pilot feasibility outcomes. We will report the numbers of eligible participants seen over the recruitment period, and the resulting rates of recruitment, compliance with randomisation, and data completion. Non completers will be characterised.

### Analysis of Secondary Outcome Measures

The pilot feasibility trial will also assess performance of potential outcome measures for a definitive trial. We will ascertain data completeness of the instruments and any potential bias in the completion of follow-up data to inform the choice of instruments in a future trial. The majority of the outcome data will be presented in simple descriptive tables presenting percentages, means and standard deviations or 5-number summary (as appropriate), for each arm of the study. This information will be used to inform the design, choice of primary outcome, necessary sample size and approach to the analysis, of the future definitive trial.

## Sample Size Calculation

The pilot feasibility trial will aim to obtain data from a minimum of 35 respondents in each trial arm at 12 month follow-up, to estimate the critical parameters to the necessary degree of precision with a continuous primary outcome, (number of occasions drinking 5+ standard drink units in a single occasion as derived from the TLFB/30).[^69^](#_ENREF_69) Assuming a pessimistic 30% loss to follow up, the sample size to be recruited will be inflated to 50 young people in each of the three arms.

# ECONOMIC EVALUATION

## Overview

The purpose of a definitive economic evaluation is to assess benefits as well as costs to patients and their families/carers, the NHS and personal social services. Within the pilot feasibility study we will develop the tools and assess the pilot feasibility of methods necessary to capture the effects of the intervention on health outcomes and associated direct costs. Specifically, we will develop data collection forms and questionnaires to capture use of hospital, primary care and social services and criminal justice resources as well as participant/family/carer costs during the follow-up period. The forms will be tailored to reflect the needs of the study participants and to ensure that there is no double counting of the use of services. For this part of the health economic analyses, data will not be statistically analysed, but rather summarised descriptively to assess the pilot feasibility of data collection instruments.

The findings from the pilot feasibility trial will then be incorporated in a mathematical decision model to provide preliminary estimates of cost, effectiveness and relative cost-effectiveness and assess the value of information (VoI) for a definitive study. It will be conducted from a societal perspective and estimate outcomes and average resource use and associated costs per participant for each trialled intervention. Results from the VoI analysis will provide additional evidence to make the economic case for funding a definitive trial and to assist in the design of that trial.

## Methods

### Data Collection

Data on health consequences will be collected by means of participant completed questionnaires collected at baseline and 12 months follow-up to capture changes in health-related quality of life outcomes (measured by EQ-5D-5L).

The associated costs will relate to resources required to provide the intervention and usage of NHS, public and personal social services as well as patient cost during the follow-up period. Within the pilot feasibility study we will compare different cost assessment tools. Questionnaires will be tested for their appropriateness to collect relevant information, which will be assessed by the amount of missing data. Open-format case diaries for staff will be used to inform on the type of activities and associated time required to deliver the interventions. These diaries will be used to produce tailored data collection tools for the definitive trial which are as parsimonious as possible.

### Data Analyses

The initial stage of value of information analysis will be the construction of a mathematical decision model that will synthesise the best available existing evidence from the literature and information from the pilot feasibility trial in order to provide preliminary estimates of effects, costs and cost-effectiveness. Uncertainty around estimates of effects, costs and cost-effectiveness will be accounted for by probabilistic sensitivity analysis and estimated using Monte Carlo simulation, as cost data are unlikely to be normally distributed. Statistical imprecision will be presented as confidence intervals around differences in effectiveness, costs and cost-effectiveness. Threshold sensitivity analyses will be applied to identify the range for costs and effects in which a treatment might need to exist in to be considered cost-effective with respect to standard thresholds for cost-effectiveness (e.g. £20,000 per QALY).

We anticipate that the results of the analysis described above will be insufficient to inform practice because of the amount of uncertainty that surrounds estimates. The VoI analysis will build upon the results of the probabilistic sensitivity analysis to estimate the expected value of sampling information (EVSI). The EVSI will quantify the value of reducing uncertainty via collection of additional data in a definitive trial. Comparing the value of additional information with the financial and opportunity cost to generate the additional information, the expected net gain (ENG) can be calculated as difference between the EVSI and total cost of conducting the further research. The optimal design of a definitive trial will maximise the expected net gain (ENG) to society with regard to sample size. The optimal sample size is that which maximises the difference between EVSI and expected total cost of the research (both the direct cost of the research itself and the opportunity cost of delaying the implementation of a worthwhile intervention whilst research is on-going). The estimated optimal sample size will be inflated according to the rate of missing data in the trial data to achieve the optimal sample size of complete data cases and to determine the final sample size for a definitive trial.

As with any method of estimating sample sizes there will be a number of uncertainties (e.g. the number of people who might benefit from one of the behavioural interventions over the expected time that the therapy might be used before becoming obsolete). For the VoI analysis we will explore these and estimate the impact on sample size estimates.

# DATA HANDLING

## Data Collection Tools and Source Document Identification

Baseline data will be collected after informed consent and will record demographics, placement type, drug and alcohol usage (AUDIT/Alcohol, Smoking and Substance Involvement Screening Tool (ASSIST) [^52^](#_ENREF_52)), mental health and well-being (baseline SDQ and WEMWBS) and health-related quality of life (EQ-5D-5L). These data will be collected electronically on tablet computers and transferred to the university server.

Follow up questionnaires will comprise:

- The 7 and 30 day Timeline Follow-back (TLFB).[^70^](#_ENREF_70) The TLFB-30 (and 7 day) will be compared to the shorter AUDIT, ASSIST and CRAFFT tools[^39^](#_ENREF_39)
- Mental health and wellbeing measured using the Strength and Difficulties Questionnaire (SDQ) and the Warwick-Edinburgh Mental Well-being scale (WEMWBS). [^40^](#_ENREF_40)^,^ [^41^](#_ENREF_41)
- Health related quality of life measured using the EQ-5D-5L.[^42^](#_ENREF_42)
- Self-reported occasions of ‘drunkenness’ in the last 30 days (past 30-day intoxication in ESPAD[^7^](#_ENREF_7)) to compare against the objective standard-drink unit measure.
- Placement stability for the young person – as part of the 12 month follow up questionnaire, questions will be developed and administered around placement stability. In addition consent will be obtained to link to social services records to assess longer term placement stability.
- Sexual behaviours measured using items taken from the computer assisted self-interview (CASI) questionnaire used in the Avon Longitudinal Study of Parents and Children (ALSPAC) supplemented with questions relating to regret in sexual encounters and unprotected sex used in the ESPAD.[^7^](#_ENREF_7)^,^ [^43^](#_ENREF_43)
- A questionnaire on antisocial and criminal behaviour used in ALSPAC. [^44^](#_ENREF_44)
- A bespoke questionnaire on use of health and personal social services.

All questionnaires will be developed during the formative research phase. The baseline and follow up questionnaires will be considered source data. Qualitative, economic and process data will also be considered as source.

## Data Handling and Record Keeping

Data from the questionnaires will be entered electronically and transferred to the Newcastle University Server.

## Access to Data

Direct access may be granted to representatives of the Sponsor, Host Institution or REC for monitoring or auditing purposes.

## Archiving

The Trial Master File (with identifiable data) will be archived by the CI in the University of Newcastle IHS for 10 years. Anonymised individual patient data is made available for sharing at the end of the study. Archiving will be authorised by the Sponsor following submission of the end of study report. The CI will request authorisation from the Sponsor to destroy the documentation at the end of the archiving period.

# MONITORING, AUDIT & INSPECTION

The Trial coordinator completing the monitoring will be independent of the Sponsor and the study team. All monitoring findings will be reported and followed up with the appropriate persons in a timely manner.

The trial may be subject to audit by representatives of the Sponsor. Data from each local authority site will be stored in the central research centre in Newcastle, who will permit trial-related audits, including access to all essential and source data relating to the trial.

A Trial Management Group will be established comprising the Chief Investigator, Senior Co-applicants, Study Statistician, Health Economist, Senior Trial Manager and Research Associates.

A Trial Oversight Committee (TOC) will be established to provide overall supervision of the trial, this group will be approved by the NIHR.

Observers from the NIHR PHR programme and from Sponsor will be invited to all TOC meetings. The committee will meet on three occasions – at the beginning of the study, and at the end of Phases 1 and 2.

# ETHICAL AND REGULATORY CONSIDERATIONS

## Research Ethics Committee Review and Reports

The CI will obtain a favourable ethical opinion from an NHS Research Ethics Committee (REC) prior to the start of the trial. Though the study does not recruit from NHS centres, due to the vulnerability of this population and the nature of the difficulties experienced i.e. drug and alcohol difficulties, NHS ethical review will be sought. All parties will conduct the trial in accordance with this ethical opinion.

The CI will notify the REC of all required substantial amendments to the trial and those non-substantial amendments that result in a change to trial documentation (e.g. protocol or patient information sheet). Substantial amendments that require a REC favourable opinion will not be implemented until this REC favourable opinion is obtained. The Sponsor will notify the REC of any serious breaches of GCP or the protocol, urgent safety measures or USARs that occur during the trial.

An annual progress report will be submitted each year to the REC by the CI until the end of the trial. This report will be submitted within 30 days of the anniversary date on which the original favourable ethical opinion was granted.

The CI will notify the REC of the early termination or end of trial in accordance with the required timelines.

## Peer Review

The study has been peer reviewed as part of the NIHR review and funding system.

## Public and Patient Involvement

Three groups of LAC (n=11 aged 12-20 years) have been consulted to develop this proposal and members are interested in developing an ongoing study PPI group. As a result of our PPI, we have changed the target age range of the study from 13-17 to 12-20 years so we do not exclude early substance users or LAC as they transition to adult services. The LAC felt it essential to involve young people in the development of the interventions and felt this research was important, especially with the rise of 'legal highs'. We also consulted widely with services including drug and alcohol workers, social workers and managers. In response to this work, we have amended our strategy for recruiting LAC into the study. LAC and professionals felt that social workers rather than LAC nurses were best placed to screen for substance use.

All our consent and information forms have been shared with young people (LAC and the Young people's advisory group (YPAG) (n=29 aged 12-18). The PPI group will meet regularly during the study and feed into the oversight group.

## Regulatory Compliance

The trial will be conducted in accordance with the Research Governance Framework. Before any site can enrol patients into the trial, that site must have received permission from the site management organisation, in this case the local authority.

## Protocol Compliance

Prospective, planned deviations or waivers must not be used without prior authorisation by the Sponsor. Any protocol deviations identified during the course of the study will be reported as per the NCTU Standard Operating Procedure. Frequently occurring deviations could be considered a serious breach.

## Notification of Serious Breaches to GCP and/or the Protocol

A serious breach is a breach which is likely to effect to a significant degree –

1. the safety or physical or mental integrity of the subjects of the trial; or
2. the scientific value of the trial

The Sponsor must be notified immediately of any incident that may be classified as a serious breach. The [Sponsor/CI] will notify the NHS REC within the required timelines in accordance with the NCTU SOP.

## Data Protection and Patient Confidentiality

All investigators must comply with the requirements of the Data Protection Act 1998 with regards to the collection, storage, processing and disclosure of personal information and will uphold the Act’s core principles.

Researcher administered questionnaires completed by participants online will be identified by the unique study identification code and initials. Only members of the research team will be able to link the unique study identification code to patient identifiable data needed for record linkage and participant contact.

All study records and Investigator Site Files will be kept at site in a locked filing cabinet with restricted access. The Trial Master file will be stored in the Institute of Health and Society, Newcastle University in a locked filing cabinet.

## Indemnity

The Sponsor (Newcastle University) will provide indemnity in the event that trial participants suffer negligent harm due to the management of trial.

The substantial employers of the protocol authors (Newcastle University) will provide indemnity in the event that trial participants suffer negligent harm due to the design of the trial.

The study sites (drug and alcohol service providers) will provide indemnity, as part of their service delivery, in the event that trial participants suffer negligent harm due to the provision of interventions as part of clinical care at their site. Service providers will meet their own service requirements for safety and clinical care.

## Amendments

It is the responsibility of the Research Sponsor to determine if an amendment is substantial or not and study procedures must not be changed without the mutual agreement of the CI, Sponsor and the Trial Oversight Committee.

Substantial amendments will be submitted to the REC and will not be implemented until this approval is in place. It is the responsibility of the CI to submit substantial amendments.

Non-substantial amendments may be made at any time with a record of the amendment held in the Trial Master File. Any non-substantial amendment that requires an update to the trial documentation will be submitted to the NHS REC for acknowledgement of the revised version of the document.

Substantial amendments and those minor amendments which may impact sites will be discussed with the LAC Lead in each local authority. Amendment documentation will be provided to sites by the CI.

## Post-Trial Care

Following the end of the study all LAC will return to usual care.

## Access to the Final Trial Dataset

The TOC will have access to the final full trial dataset if requested. Other researchers that wish to access the data will need permission from the CI, TOC and NIHR.

# DISSEMINATION POLICY

Data arising from the study will be the property of the Chief Investigator and Co-Investigators. Publication will be the responsibility of the Chief Investigator and published under the authorship agreed with the Co-Investigators.

On completion of the study, data will be analysed and tabulated and a final study report prepared. This will be available from the NIHR PHR and FUSE websites.

In addition to the NIHR monograph, it is planned to publish this study in peer review articles and to present data at national and international meetings. Results of the study will also be reported to the Sponsor and Funder, and will be available on their web site. Publications will be shared with the TOC and funders. Individuals will not be identified from any study report. Individuals will not be identified from any study report.

Participants will be informed about their treatment and their contribution to the study at the end of the study, including a lay summary of the results. This will be developed by the study PPI group.

Publication of the results of the study will follow NIHR guidance on communicating research outcomes. NIHR will also receive full citations of research outputs when these become available.

All research reports issued by individual researchers and/or research teams will:

● Credit the NIHR as a funding organisation

● Carry the NIHR disclaimer

Only anonymised data will be used when publishing results; no personal identifiers will be used

# REFERENCES

1. Hale DR, Viner RM. Policy responses to multiple risk behaviours in adolescents. *Journal of public health* 2012; **34 Suppl 1**: i11-9.

2. Rehm J, Taylor B, Room R. Global burden of disease from alcohol, illicit drugs and tobacco. *Drug and alcohol review* 2006; **25**(6): 503-13.

3. Department of Health England. Alcohol and drugs prevention, treatment and recovery: why invest?, 2013.

4. Hodgins S, Larm P, al e. Multiple adverse outcomes over 30 years following adolescent substance misuse treatment. *Acta Psychiat Scand* 2009; **119**(6): 484-93.

5. Chen CY, Storr CL, Anthony JC. Early-onset drug use and risk for drug dependence problems. *Addictive behaviors* 2009; **34**(3): 319-22.

6. Gilvarry E. Substance abuse in young people. *J Child Psychol Psyc* 2000; **41**(1): 55-80.

7. Hibell B. The 2011 ESPAD report : substance use among students in 36 Europeans countries. Luxembourg: Publications Office of the European Union; 2012.

8. Elizabeth Fuller (Editor). Smoking, drinking and drug use among young people in England in 2012: Health and Social Care Information Centre,, 2012.

9. Faculty of Addictions Psychiatry. Royal College of Psychiatrists. One new drug a week: Why novel psychoactive substances and club drugs need a different response from UK treatment providers,, 2014.

10. Vickerstaff J. Statistical first release: Children looked after in England (including adoption and care leavers) year ending 31 March 2014. Department for Education National statistics, 2014.

11. National Institute of Clinical Excellence. Interventions to reduce substance misuse among vulnerable young people: Evidence Update April 2014, 2014.

12. The National Treatment Agency for Substance Misuse. Substance misuse amongst young people 2011-2012, 2012.

13. Professor Dame Sally Davies. Chief Medical Officer's annual report 2012: Our Children Deserve Better: Prevention Pays, 2012.

14. Simkiss D. Looked after Children and Care Leavers. In: Davis DS, ed. Annual Report of the Chief Medical Officer 2012, Our Children Deserve Better: Prevention Pays.

15. Simkiss DE, Spencer NJ, Stallard N, Thorogood M. Health service use in families where children enter public care: a nested case control study using the General Practice Research Database. *BMC health services research* 2012; **12**: 65.

16. Simkiss DE, et al. A systematic literature review of the risk factors associated with children entering public care. *Child: care, health and development* 2013; **39**(5): 628-42.

17. Meltzer H. The mental health of young people looked after by local authorities in England. London: H.M.S.O.; 2003.

18. Ward J, Henderson Z, Pearson G. One problem among many : drug use among care leavers in transition to independent living. London: Home Office, Research, Development and Statistics Directorate; 2003.

19. Blyth L. Outcomes for Children Looked After by Local Authorities in England, as of 31 March 2012: Office of National Statistics, 2012.

20. Ford T, Vostanis P, Meltzer H, Goodman R. Psychiatric disorder among British children looked after by local authorities: comparison with children living in private households. *The British journal of psychiatry : the journal of mental science* 2007; **190**: 319-25.

21. Viner RM, Taylor B. Adult health and social outcomes of children who have been in public care: Population-based study. *Pediatrics* 2005; **115**(4): 894-9.

22. Broderick R, McCoard S, Carnie J. Prisinors who have been in care as "Looked after Children and Care Leavers": Scottish Prison Service, 2014.

23. Foxcroft DR, Tsertsvadze A. Universal family-based prevention programs for alcohol misuse in young people. *The Cochrane database of systematic reviews* 2011; (9): CD009308.

24. Foxcroft DR, Tsertsvadze A. Universal multi-component prevention programs for alcohol misuse in young people. *The Cochrane database of systematic reviews* 2011; (9): CD009307.

25. Foxcroft DR, Tsertsvadze A. Universal school-based prevention programs for alcohol misuse in young people. *The Cochrane database of systematic reviews* 2011; (5): CD009113.

26. Carney T, Myers B. Effectiveness of early interventions for substance-using adolescents: findings from a systematic review and meta-analysis. *Substance abuse treatment, prevention, and policy* 2012; **7**: 25.

27. Foxcroft DR, Coombes L, Wood S, Allen D, Almeida Santimano NM. Motivational interviewing for alcohol misuse in young adults. *The Cochrane database of systematic reviews* 2014; **8**: CD007025.

28. Segrott J, Gillespie D, Holliday J, et al. Preventing substance misuse: study protocol for a randomised controlled trial of the Strengthening Families Programme 10-14 UK (SFP 10-14 UK). *BMC public health* 2014; **14**: 49.

29. Tripodi SJ, Bender K, et al. Interventions for reducing adolescent alcohol abuse: a meta-analytic review. *Archives of pediatrics & adolescent medicine* 2010; **164**(1): 85-91.

30. Mirza K, Mirza S. Adolescent substance misuse. *Psychiatry* 2008; **7**(8): 357-62.

31. Dixon J. Young people leaving care : a study of costs and outcomes : report to the Department for Education & Skills. York: The University of York, Social Work Research & Development Unit; 2006.

32. Miller WR ZA, Carlo DSW, DiClemente C, Rychtarik RG,. Motivational Enhancement Therapy Manual: A Clinical Research Guide for Therapists Treating Individuals With Alcohol Abuse and Dependence: NIH publication 1999.

33. Copello A, Orfor J, Hodgson R, Tober G, Barrett C, Ukatt Research Team. United Kingdom Alcohol Treatment Trial. Social behaviour and network therapy basic principles and early experiences. *Addictive behaviors* 2002; **27**(3): 345-66.

34. Tevyaw T, Monti P. Motivational enhancement and other brief interventions for adolescent substance abuse: foundations, applications and evaluations. *Addiction* 2004; **99 (2)**: 63-75.

35. Newbury-Birch D, Gilvarry E, McArdle P, et al. The impact of alcohol consumption on young people: A review of reviews. Department of Children Schools and Families; 2009.

36. UKATT Research Team. Effectiveness of treatment for alcohol problems: findings of the randomised UK alcohol treatment trial (UKATT). *BMJ* 2005; **331**(7516): 541.

37. Dennis ML, Funk R, et al. Cross-validation of the alcohol and cannabis use measures in the Global Appraisal of Individual Needs (GAIN) and Timeline Followback (TLFB; Form 90) among adolescents in substance abuse treatment. *Addiction* 2004; **99 ( 2)**: 120-8.

38. Nutt DJ, King LA, Phillips LD, Independent Scientific Committee on D. Drug harms in the UK: a multicriteria decision analysis. *Lancet* 2010; **376**(9752): 1558-65.

39. Knight J, Sherritt L, Harris S, Gates E, Chang G. Validity of Brief Alcohol Screening Tests Among Adolescents: A Comparison of the AUDIT, POSIT, CAGE and CRAFFT. *Alcoholism: Clinical and Experimental Research* 2003; **27**(1): 67-73.

40. Goodman R. Psychometric properties of the strengths and difficulties questionnaire. *J Am Acad Child Adolesc Psychiatry* 2001; **40**(11): 1337-45.

41. Clarke A, Friede T, Putz R, et al. Warwick-Edinburgh Mental Well-being Scale (WEMWBS): validated for teenage school students in England and Scotland. A mixed methods assessment. *BMC public health* 2011; **11**: 487.

42. Janssen MF, Pickard AS, Golicki D, et al. Measurement properties of the EQ-5D-5L compared to the EQ-5D-3L across eight patient groups: a multi-country study. *Quality of life research : an international journal of quality of life aspects of treatment, care and rehabilitation* 2013; **22**(7): 1717-27.

43. Waylen A E, Ness A, McGovern P, Dieter Wolke D, Low N. Romantic and Sexual Behavior in Young Adolescents: Repeated Surveys in a Population-Based Cohort. *Journal of Early Adolescence* 2010; **30**(3): 432-43.

44. Cho SB, Heron J, Aliev F, et al. Directional relationships between alcohol use and antisocial behavior across adolescence. *Alcohol Clin Exp Res* 2014; **38**(7): 2024-33.

45. Smith DJ, McVie S. Edinburgh study of youth transitions and crime. *British Journal of Criminology* 2003; **43**: 169-95.

46. Lingam R, Gupta P, Zafar S, et al. Understanding care and feeding practices: building blocks for a sustainable intervention in India and Pakistan. *Annals of the New York Academy of Sciences* 2014; **1308**: 204-17.

47. Yardley L, Morrison L, Bradbury K, Muller I. The person-based approach to intervention development: application to digital health-related behavior change interventions. *Journal of medical Internet research* 2015; **17**(1): e30.

48. Donovan J, Sanders C. Key issues in the analysis of qualitative data in health services research. In: Bowling A, Ebrahim S, eds. Handbook of health research methods : investigation, measurement and analysis. Maidenhead: Open University Press; 2005: 515 - 32.

49. Bourdieu P. Structures, habitus, practices. The logic of practice Cambridge; 1990: 52-65.

50. Scott S, Kaner E. Alcohol and Public Health: Heavy drinking is a heavy price to pay for populations. . *Journal of public health* 2014; **36**(3): 396-8.

51. Hoffmann TC, et al. Better reporting of interventions: template for intervention description and replication (TIDieR) checklist and guide. *BMJ* 2014; **348**: g1687.

52. Gryczynski J, Kelly SM, Mitchell SG, Kirk A, O'Grady KE, Schwartz RP. Validation and performance of the Alcohol, Smoking and Substance Involvement Screening Test (ASSIST) among adolescent primary care patients. *Addiction* 2015; **110**(2): 240-7.

53. Kirby D. BDI Logic Models: A Useful Tool for Designing, Strengthening and Evaluating Programmes to Reduce Adolescent Sexual Risk-Taking, Pregnancy, HIV and Other STDs., 2004.

54. Lundahl B W, et al. A Meta-Analysis of Motivational Interviewing: Twenty-Five Years of Empirical Studies. *Research on Social Work Practice* 2010; **20**(2): 137-60.

55. Rollnick S, Miller WR, Butler C. Motivational interviewing in health care : helping patients change behavior. New York ; London: Guilford; 2008.

56. Copello A. Social behaviour and network therapy for alcohol problems. London: Routledge; 2009.

57. Beattie MC, Longabaugh R. General and alcohol-specific social support following treatment. *Addictive behaviors* 1999; **24**(5): 593-606.

58. Havassy BE, Hall SM, Wasserman DA. Social support and relapse: commonalities among alcoholics, opiate users, and cigarette smokers. *Addictive behaviors* 1991; **16**(5): 235-46.

59. Wasserman DA, Stewart AL, Delucchi KL. Social support and abstinence from opiates and cocaine during opioid maintenance treatment. *Drug and alcohol dependence* 2001; **65**(1): 65-75.

60. UKATT Research Team. Cost effectiveness of treatment for alcohol problems: findings of the randomised UK alcohol treatment trial (UKATT). *BMJ* 2005; **331**(7516): 544.

61. Watson J., Back D., Toner P., et al. A randomised controlled pilot feasibility trial of family and social network intervention for young people who misuse alcohol and drugs: study protocol (Y-SBNT). Pilot and Pilot feasibility Studies. 2015; **1**(8).

62. Steckler AB, Linnan L. Process evaluation for public health interventions and research. San Francisco ; [Great Britain]: Jossey-Bass; 2002.

63. Carroll C, Patterson M, Wood S, Booth A, Rick J, Balain S. A conceptual framework for implementation fidelity. *Implementation science : IS* 2007; **2**: 40.

64. Tober G, Clyne W, Finnegan O, Farrin A, Russell I, Team UR. Validation of a scale for rating the delivery of psycho-social treatments for alcohol dependence and misuse: the UKATT Process Rating Scale (PRS). *Alcohol and alcoholism* 2008; **43**(6): 675-82.

65. May CR, Mair F, Finch T, et al. Development of a theory of implementation and integration: Normalization Process Theory. *Implementation science : IS* 2009; **4**: 29.

66. Denzin NK. The research act : a theoretical introduction to sociological methods. 3rd ed. ed. Englewood Cliffs, N.J.: Prentice Hall; 1989.

67. Farmer T, Robinson K, Elliott SJ, Eyles J. Developing and implementing a triangulation protocol for qualitative health research. *Qualitative health research* 2006; **16**(3): 377-94.

68. O'Cathain A, Murphy E, Nicholl J. Three techniques for integrating data in mixed methods studies. *BMJ* 2010; **341**: c4587.

69. Teare MD, Dimairo M, Shephard N, Hayman A, Whitehead A, Walters SJ. Sample size requirements to estimate key design parameters from external pilot randomised controlled trials: a simulation study. *Trials* 2014; **15**: 264.

70. Hjorthoj CR, Hjorthoj AR, Nordentoft M. Validity of Timeline Follow-Back for self-reported use of cannabis and other illicit substances--systematic review and meta-analysis. *Addictive behaviors* 2012; **37**(3): 225-33.

# APPENDICES

## Appendix 1 - Safety Reporting Diagram

| Adverse Event | | | | | | |
| --- | --- | --- | --- | --- | --- | --- |
| Not Serious | | Not sure |  | Serious | |  |
| Unrelated | Related |  | Unrelated | | Related | |
|  |  |  |  | | Expected | Unexpected |
| Complete  CRF | Complete  CRF | Complete SAE form | Complete SAE form | | Complete SAE form | Notify [Sponsor/CI] of USAR |

Contact details for reporting SAEs and USARs

Please send SAE form(s) via [Fax number]

Or

[Telephone number] [Availability]

## Appendix 2 – Amendment History

##

| **Amendment Number** | **Protocol version no.** | **Date issued** | **Author(s) of changes** | **Details of changes made** |
| --- | --- | --- | --- | --- |
|  |  |  |  |  |

{Enter all amendments to the protocol here whether substantial or non-substantial. Substantial amendments will require approval by the NHS REC. Non-substantial amendments should be sent to the NHS REC for acknowledgement only}
